# Cost-effective mitigation of nitrogen pollution from global croplands

**DOI:** 10.1038/s41586-022-05481-8

**Published:** 2023-01-04

**Authors:** Baojing Gu, Xiuming Zhang, Shu Kee Lam, Yingliang Yu, Hans J. M. van Grinsven, Shaohui Zhang, Xiaoxi Wang, Benjamin Leon Bodirsky, Sitong Wang, Jiakun Duan, Chenchen Ren, Lex Bouwman, Wim de Vries, Jianming Xu, Mark A. Sutton, Deli Chen

**Affiliations:** 1 College of Environmental and Resource Sciences, Zhejiang University, Hangzhou, China; 2 Policy Simulation Laboratory, Zhejiang University, Hangzhou, China; 3 School of Agriculture and Food, The University of Melbourne, Melbourne, Victoria Australia; 4 Key Laboratory of Agricultural Environment of the Lower Reaches of the Yangtze River, Institute of Agricultural Resources and Environment, Jiangsu Academy of Agricultural Sciences, Nanjing, China; 5 PBL Netherlands Environmental Assessment Agency, The Hague, The Netherlands; 6 School of Economics and Management, Beihang University, Beijing, China; 7 International Institute for Applied Systems Analysis, Laxenburg, Austria; 8 China Academy for Rural Development, Zhejiang University, Hangzhou, China; 9 Department of Agricultural Economics and Management, School of Public Affairs, Zhejiang University, Hangzhou, China; 10 Potsdam Institute for Climate Impact Research (PIK), Potsdam, Germany; 11 Department of Earth Sciences - Geochemistry, Faculty of Geosciences, Utrecht University, Utrecht, The Netherlands; 12 Environmental Systems Analysis Group, Wageningen University & Research, Wageningen, The Netherlands; 13 Zhejiang Provincial Key Laboratory of Agricultural Resources and Environment, Zhejiang University, Hangzhou, China; 14 Edinburgh Research Station, UK Centre for Ecology & Hydrology, Penicuik, UK

**Keywords:** Element cycles, Agriculture

## Abstract

Cropland is a main source of global nitrogen pollution^1,2^. Mitigating nitrogen pollution from global croplands is a grand challenge because of the nature of non-point-source pollution from millions of farms and the constraints to implementing pollution-reduction measures, such as lack of financial resources and limited nitrogen-management knowledge of farmers^3^. Here we synthesize 1,521 field observations worldwide and identify 11 key measures that can reduce nitrogen losses from croplands to air and water by 30–70%, while increasing crop yield and nitrogen use efficiency (NUE) by 10–30% and 10–80%, respectively. Overall, adoption of this package of measures on global croplands would allow the production of 17 ± 3 Tg (10^12^ g) more crop nitrogen (20% increase) with 22 ± 4 Tg less nitrogen fertilizer used (21% reduction) and 26 ± 5 Tg less nitrogen pollution (32% reduction) to the environment for the considered base year of 2015. These changes could gain a global societal benefit of 476 ± 123 billion US dollars (USD) for food supply, human health, ecosystems and climate, with net mitigation costs of only 19 ± 5 billion USD, of which 15 ± 4 billion USD fertilizer saving offsets 44% of the gross mitigation cost. To mitigate nitrogen pollution from croplands in the future, innovative policies such as a nitrogen credit system (NCS) could be implemented to select, incentivize and, where necessary, subsidize the adoption of these measures.

## Abstract (web summary)

A meta-analysis of 1,521 field observations from the past two decades led to the identification of 11 key measures to cost-effectively mitigate nitrogen pollution from global croplands.

## Main

Feeding the growing and increasingly wealthy global population causes huge pressures on food and animal feed production^4^. To increase food and feed supply, intensified agriculture has used more and more nitrogen (N) fertilizers and manure^2^. However, more than half of these N inputs to croplands are lost to air and water, close to the annual total chemical N fertilizer of 120 Tg used globally, leading to severe air pollution (especially fine particle matter, PM_2.5_), water pollution (especially eutrophication), soil acidification, climate change, ozone depletion in the stratosphere and biodiversity loss^1,5^. Therefore, reducing N loss from croplands can not only increase direct economic returns from a lower requirement for fertilizers but also improve human health and ecosystem services and reduce climate change^6^.

Mitigation of N pollution from croplands has attracted global attention^7^. Best management practices have been developed, such as the 4R nutrient stewardship (right fertilizer type, right amount, right placement and right time) and soil testing to precisely apply fertilizer to the soil^8,9^. However, these management practices are seldom fully implemented owing to many constraints, such as a high heterogeneity of best practices on the local scale and, in some cases, high implementation costs for farmers (including high capital and/or running costs)^2,10^. Thus, we identified a package of the most effective on-farm field mitigation measures to abate N pollution and estimated the costs and benefits to facilitate the implementation of these measures. We first screened the performance of available measures through a global meta-analysis and quantified their potential to abate N pollution in global croplands. Then we calculated the implementation cost of these measures and their social benefits. Finally, we quantified the consequence of applying these measures across global regions, dividing the measures into three tiers, differing in implementation challenges, thus affecting their estimated regional applicability.

## Mitigation potential for the year 2015

**Fig. 1:**
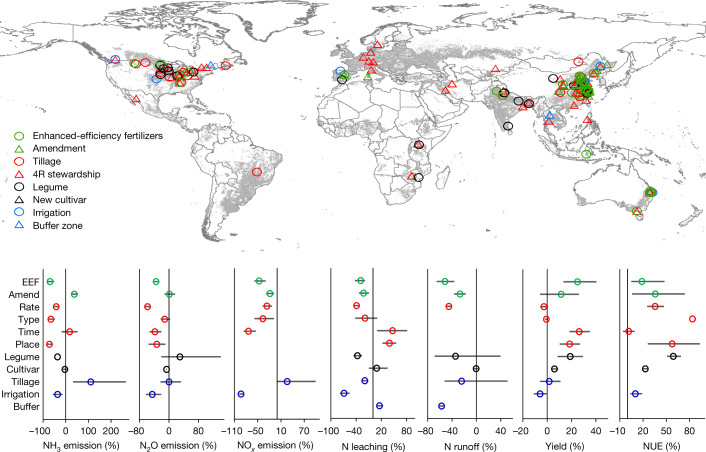
Geographical distribution of the sample sites of the global meta-analysis and the effects of management practices on cropland N use and loss. 4R stewardship refers to the right fertilizer type, right amount, right placement and right time on fertilization; EEF refers to enhanced-efficiency fertilizers; amendment refers to amendment applied to croplands such as biochar; tillage refers to change from tillage to no tillage; legume refers to rotation of legumes with other crops; irrigation refers to drip irrigation or optimal irrigation; buffer zone refers to the use of wetlands or marginal lands between croplands and rivers. There would be other effective measures excluded in this study owing to the lack of studies or beyond croplands, such as manure management. Measures on N abatement may interact, which was not considered in this study owing to limited studies. The colours in the meta-analysis refer to different types of measure: additive (green), 4R nutrient stewardship (red), crop species (black) and biophysical management (blue). The number of observations in this figure is listed in Supplementary Figs. 4–14. The base map is applied without endorsement from GADM data (https://gadm.org/). Source data

Through conducting a meta-analysis of 1,521 field observations in the past two decades, we identified a group of 11 key measures that can mitigate N losses from croplands across global regions (Fig. 1 and Supplementary Figs. 5–14), being selected according to criteria such as having detailed information on field experiments (see Methods). The measures were divided into three tiers based on expert judgement (Supplementary Fig. 2): (1) Tier 1 including N addition approaches (enhanced-efficiency fertilizers (EEFs), organic amendments including manure and straw), crop legume rotation and application of buffer zones; (2) Tier 2 being the 4R nutrient stewardship, that is, the right rate, type, time and place of fertilizer application; and (3) Tier 3 being the introduction of new cultivars, optimal irrigation and tillage (Supplementary Fig. 2). The implementation barriers and costs of the mitigation measures vary substantially. Our categorization of mitigation measures according to the three tiers allows us to recognize increasing implementation challenges from lower to higher tiers (Supplementary Table 1 and Supplementary Fig. 2). The lower the tier, the easier and cheaper it is for farmers to adopt the measure, as it requires limited knowledge and little extra effort (see Methods, ‘Tier classification of mitigation measures’).

Manure management in feedlots is not included because it does not belong to cropland N management practices. However, once manure is input into croplands, it is treated as the organic amendment that can change the cropland N cycle. Implementation of these measures would reduce several N losses: emission of ammonia (NH_3_), nitrogen oxides (NO_*x*_) and nitrous oxide (N_2_O), leaching and runoff of reactive N (N_r_). In principle, some measures may decrease losses of one N species while increasing others (Fig. 1), especially because reducing N losses increases the amount of N available in the soil, indicating the need for compensatory actions, such as reducing N inputs or increasing harvests^9^.

Overall, most of the measures listed can effectively reduce total N losses by 30–70% (Supplementary Table 1 and Fig. 1), while increasing yield and NUE (harvested N divided by total N input) by 10–30% and 10–80%, respectively. According to our meta-analysis, the EEFs, 4R nutrient stewardship, irrigation and legume rotation have better overall performance in the reduction of N pollution compared with other measures (Supplementary Table 1). In reducing N losses, while increasing yield and the NUE, the mitigation measures save fertilizer N for crop use, partly also by enhancing manure N inputs. For instance, the EEFs can reduce the total N loss by 47%, while increasing crop yield by 25% and the NUE by 18%. More detailed effects of measures on crop yield, NUE and N losses are described in Supplementary Figs. 5–14.

**Fig. 2:**
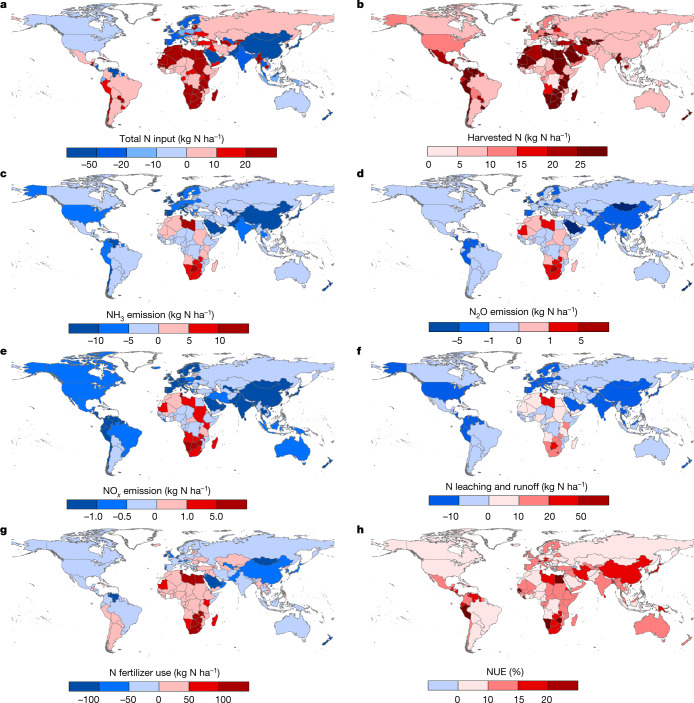
Changes in global N budget in croplands with the best adoption of the 11 selected measures. **a**, Total N input. **b**, Harvested N. **c**, NH_3_ emission. **d**, N_2_O emission. **e**, NO_*x*_ emission. **f**, N leaching and runoff. **g**, N fertilizer use. **h**, NUE. The base year is 2015 and the changes are calculated on the basis of the differences between the N fluxes in 2015 before and after the implementation of the most appropriate set of measures at the national level. The changes in NUE are in percentage points; for instance, the NUE increased by 17 percentage points in China. The base map is applied without endorsement from GADM data (https://gadm.org/). Source data

**Extended Data Fig. 1:**
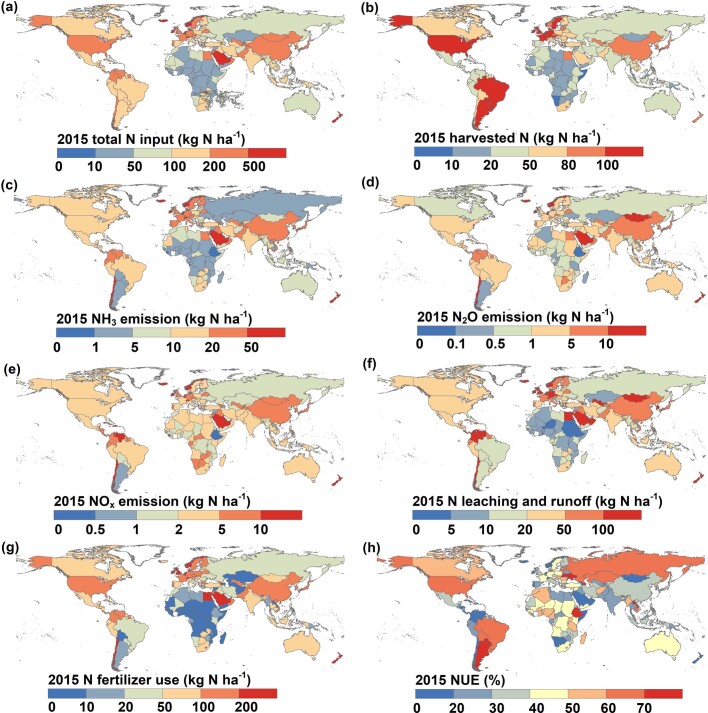
N budget of croplands on the country scale in 2015. **a**, Total N input rate. **b**, Harvested N rate. **c**, NH_3_ emission rate. **d**, N_2_O emission rate. **e**, NO_*x*_ emission rate. **f**, N leaching and runoff rate. **g**, N fertilizer rate. **h**, NUE. The base map is applied without endorsement from GADM data (https://gadm.org/). The calculation is based on the CHANS model. Source data

**Extended Data Fig. 2:**
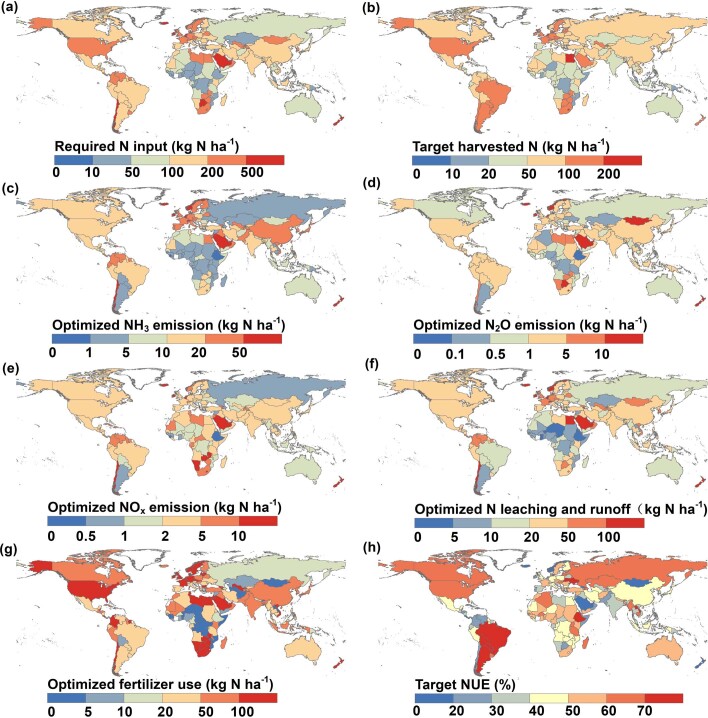
N budget of croplands on the country scale in 2015 under optimized N management. **a**, Total N input rate. **b**, Harvested N rate. **c**, NH_3_ emission rate. **d**, N_2_O emission rate. **e**, NO_*x*_ emission rate. **f**, N leaching and runoff rate. **g**, N fertilizer rate. **h**, Target NUE. Here we assume that the optimal N management could enable the cropland to maintain the amount of harvested N while achieving the target NUE. The base map is applied without endorsement from GADM data (https://gadm.org/). The calculation is based on the optimized CHANS model. Source data

Integrating the reduction potential of the 11 different measures into the N budget models coupled human and natural systems (CHANS)^11^, the Model of Agricultural Production and its Impact on the Environment (MAgPIE)^12^ and Integrated Model to Assess the Global Environment (IMAGE)^13^, we estimated the changes in the global cropland N budget in 2015 (Extended Data Fig. 1). The model results indicate that these mitigation measures reduce 32% of N_r_ emission to the air (NH_3_, NO_*x*_ and N_2_O) and water (runoff to surface water and leaching to groundwater), by 10 ± 2 and 16 ± 4 Tg N, respectively, in 2015 (Fig. 2). We also estimate a reduction of 8 ± 3 Tg N in N_2_ emissions. As N_2_ is an unreactive gas, this reduction does not constitute environmental improvement, but together with the reduction in other forms of N lost to the environment, it can save the use of N fertilizers and reduce the upstream cost of fertilizer production, including the associated emission of pollutants and greenhouse gases (GHG)^1^. These changes would reduce the total N_r_ input to global croplands by 18 ± 4 Tg N in 2015 and increase N_r_ harvest by 17 ± 3 Tg N (20% increase), resulting in an increase of NUE from 42% to 55% globally (Extended Data Fig. 2). We calculated that these measures increase manure and straw recycling to croplands by 11 ± 2 Tg N and reduce atmospheric deposition because of less volatilized N_r_ and chemical fertilizers input to croplands by 5 ± 1 and 22 ± 4 Tg N (21% reduction), respectively.

**Extended Data Fig. 3:**
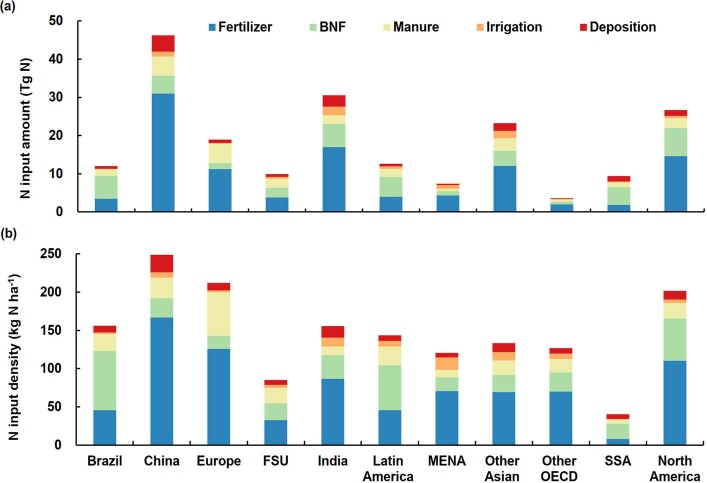
N input to croplands in 2015 in the main global regions. **a**, Total N input. **b**, Area-based N input. Deposition refers to the total atmospheric N deposition to croplands. The data were derived from the CHANS model. FSU, former Soviet Union; MENA, Middle East and North Africa; SSA, sub-Saharan Africa. Source data

**Extended Data Fig. 4:**
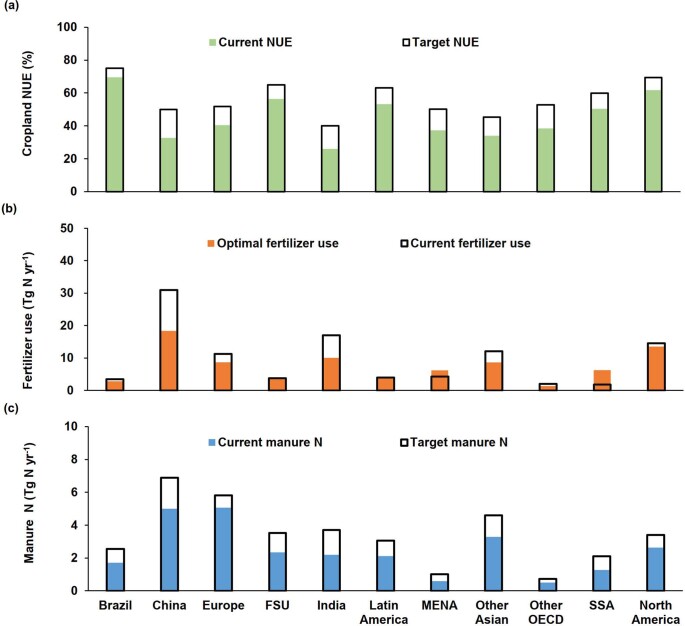
Gaps between current and optimal 2015 in terms of NUE, fertilizer use and manure application. **a**, Current NUE and target NUE in 2015. **b**, Current and optimal fertilizer N use in croplands in 2015. The optimal fertilizer use was calculated according to the balance between required N input (determined by target NUE and harvested N) and other N inputs (manure, deposition, irrigation, biological N fixation). **c**, Current and target manure N recycling to the croplands. FSU, former Soviet Union; MENA, Middle East and North Africa; OECD, Organization for Economic Co-operation and Development; SSA, sub-Saharan Africa. Source data

Reduction of N_r_ input and losses varied globally (Fig. 2). The largest reduction of N_r_ input (>50 kg N ha^−1^) and losses (>25 kg N ha^−1^) were calculated in East and South Asia and Southeast Asia, indicating overuse of N fertilizer in these global regions. We calculated lower reduction (<10 kg N ha^−1^) in high-income regions such as the European Union, Australia and North America, where N_r_ use in croplands is closer to the estimated economic optimal level, although there is still potential to further reduce N_r_ input and loss^14^. With low N_r_ input to parts of Africa, Latin America, East Europe and Middle Asia, there is potential to increase N_r_ input (>20 kg N ha^−1^) to increase food production, especially in Africa, where insufficient N inputs have depleted the soil N reserves^2^. By contrast, crop yield and NUE are expected to increase in East and South Asia owing to the optimization of N use (Fig. 2). The NUE would only slightly change in high-income countries such as the USA and low-income countries in Africa, where NUEs are already high^14^. Detailed changes in the N budget on national and regional scales can be found in Extended Data Figs. 1–4.

## Costs and benefits for the year 2015

**Fig. 3:**
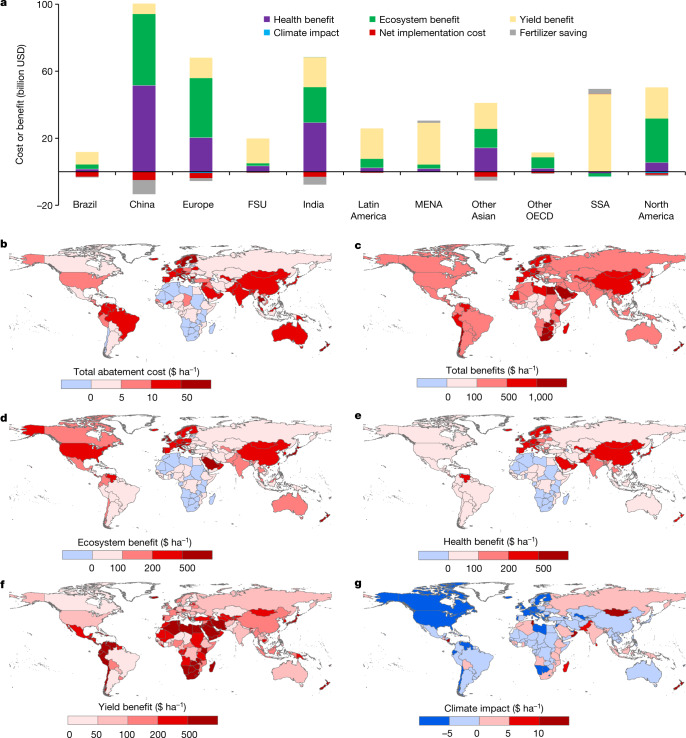
Costs and benefits of implementation of the 11 selected measures in 2015. **a**, Total costs and benefits variations in the main global regions in 2015 under best-fitted mitigation options. Negative values refer to costs and positive values refer to benefits. Implementation costs refer to the net costs for farmers to implement measures accounting for fertilizer savings. **b**, Total abatement cost. **c**, Total benefits. **d**, Ecosystem benefit. **e**, Health benefit. **f**, Yield benefit. **g**, Climate impact. All values are estimated and expressed in constant 2017 USD. FSU, former Soviet Union; MENA, Middle East and North Africa; OECD, Organization for Economic Co-operation and Development; SSA, sub-Saharan Africa. The base map is applied without endorsement from GADM data (https://gadm.org/). Source data

We estimate the net global mitigation costs of the measures derived from changes in labour cost, material cost and services at 19 ± 5 billion USD in 2015, being equal to about 14 USD ha^−1^ (Fig. 3). These costs include the net benefits from fertilizer savings, estimated at 15 ± 4 billion USD, which means that the initial implementation cost (excluding fertilizer savings) is around 34 ± 9 billion USD. Fertilizer savings thus compensate 44% of the gross implementation cost of these measures. On the basis of our estimates, China alone would require an estimated 5 ± 1 billion USD (26 USD ha^−1^) to implement the mitigation measures, followed by India, which would need 3 ± 1 billion USD (16 USD ha^−1^) (Fig. 3a); these two countries are the largest consumers of synthetic N fertilizers and emitters of N_r_ to the environment. The net mitigation cost of other countries is normally less than 1 billion USD, resulting mainly from the small amount of N_r_ loss and/or more advanced agricultural machinery and well-trained farmers, allowing low transaction costs to implement these measures. To implement the N_r_ abatement measures included here, such as the 4R nutrient stewardship, farmers probably need to change management practices on their lands^3^.

Our estimated net economic benefit to the whole of society resulting from abatement of N_r_ losses from croplands, considering benefits to crop yield, human health, ecosystems and climate change, are approximately 25 times that of the implementation cost, that is, 476 ± 123 billion USD (Fig. 3a and Supplementary Table 4). Yield increase alone is estimated to contribute 196 ± 45 billion USD, mainly in regions with low crop yields, such as the Middle East and North Africa and sub-Saharan Africa owing to insufficient use of N fertilizers, and in China and India owing to overuse of N fertilizers. Both insufficient use and overuse of N fertilizers can reduce crop yields, and measures optimizing fertilizer use thus increase crop yield and save overall fertilizer use globally.

Apart from the benefits in fertilizer savings and increased N in harvests, about 130 ± 41 billion USD stems from reduced premature mortality, most notably through avoiding respiratory diseases resulting from PM_2.5_ pollution^15^. The remaining 152 ± 36 billion USD stems from reduced damages to ecosystem services, such as reduced recreation and property value by eutrophication. Climate impacts are estimated at approximately −2 ± 1 billion USD (Fig. 3a), reflecting the potential damage to climate by improved cropland N management (Fig. 3g). According to our approach, the abatement of N_r_ emission in some regions could also aggravate global warming through reduction of carbon sequestration in natural ecosystems resulting from reduction of atmospheric N deposition derived from reduced NH_3_ emission from croplands^16^.

**Extended Data Fig. 5:**
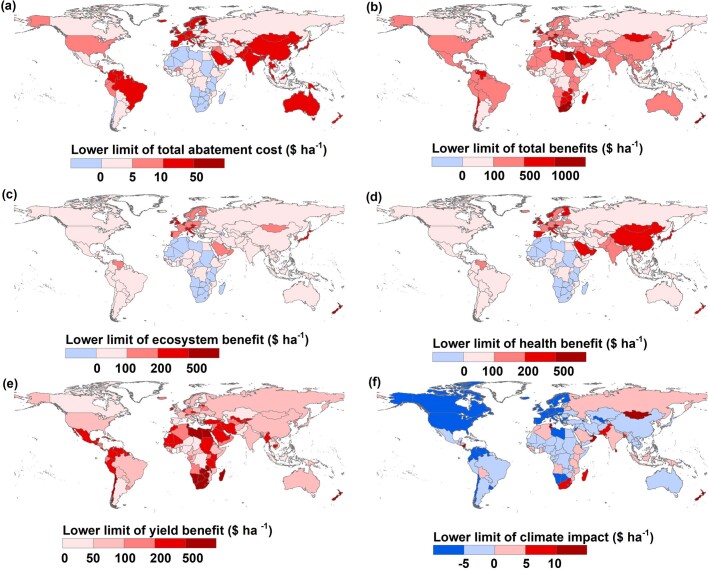
Lower limits of cost and benefit of N pollution abatement in croplands on a country scale. **a**, Total abatement cost. **b**, Total benefits. **c**, Ecosystem benefit. **d**, Health benefit. **e**, Yield benefit. **f**, Climate impact. The base map is applied without endorsement from GADM data (https://gadm.org/). Source data

**Extended Data Fig. 6:**
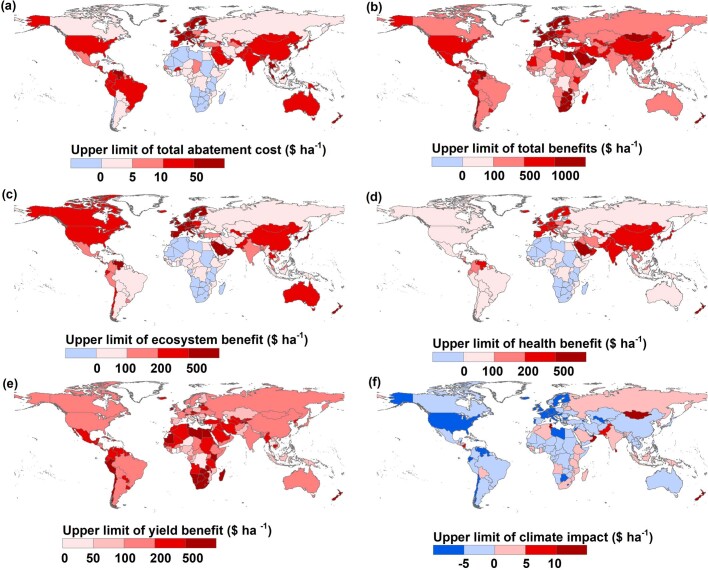
Upper limits of cost and benefit of N pollution abatement in croplands on a country scale. **a**, Total abatement cost. **b**, Total benefits. **c**, Ecosystem benefit. **d**, Health benefit. **e**, Yield benefit. **f**, Climate impact. The base map is applied without endorsement from GADM data (https://gadm.org/). Source data

These mitigation benefits are for the whole of the global society, and the high benefit-to-cost ratio provides a strong motive to implement these measures. However, these benefits might be difficult to achieve on regional and local scales, given the variations in benefit-to-cost ratios and constraints. If not taking societal benefits (human health, ecosystems and climate) into consideration, in regions with overuse of N fertilizers (‘too much’ regions) the monetized cost of yield reduction is close to the total implementation cost, which explains the lack of financial incentives to reduce fertilizer use (Fig. 3). By contrast, in ‘too little’ regions with insufficient use of N fertilizers, the low accessibility to fertilizers strongly constrains the increased use of N fertilizers despite the much higher yield benefit-to-cost ratios. More details of the uncertainties of costs and benefits of mitigating N pollution can be found in Supplementary Table 4 and Extended Data Figs. 5 and 6.

## Implementation of measures towards 2050

**Fig. 4:**
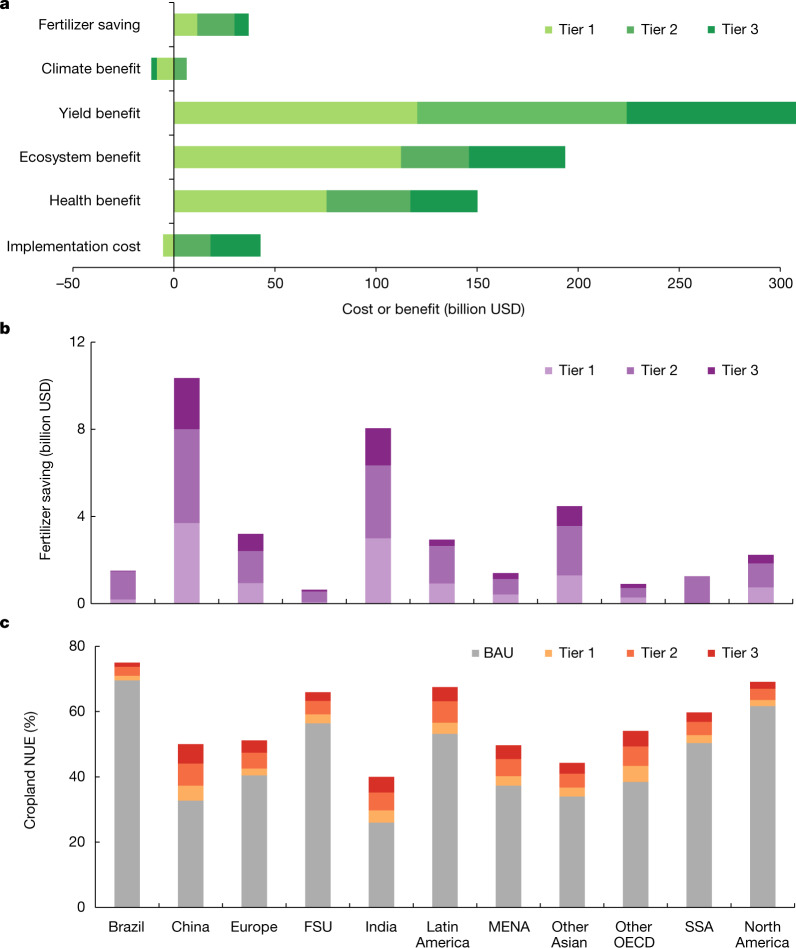
Costs and benefits of the tiered approach towards 2050 by region. **a**, Global costs and benefits. **b**, Regional fertilizer savings. **c**, Regional NUE change. BAU, business as usual; FSU, former Soviet Union; MENA, Middle East and North Africa; OECD, Organization for Economic Co-operation and Development; SSA, sub-Saharan Africa. Definitions of the different tiers can be found in Methods. The base map is applied without endorsement from GADM data (https://gadm.org/). Source data

To inform future policymaking, we explored the cropland N inputs and flows towards the year 2050 under different scenarios using the three ‘tiers’ types of mitigation measure. With the increase of N fluxes in 2050, the total global abatement cost and benefit owing to avoided damages will also increase for all three tiers. On the basis of our estimates, Tier 1 measures offer net financial benefits to farmers, whereas Tier 2 and Tier 3 measures are cost-effective to the whole of society, but involve substantial implementation costs for farmers, although they could also have financial benefits from crop yields and fertilizer savings (Fig. 4).

**Fig. 5:**
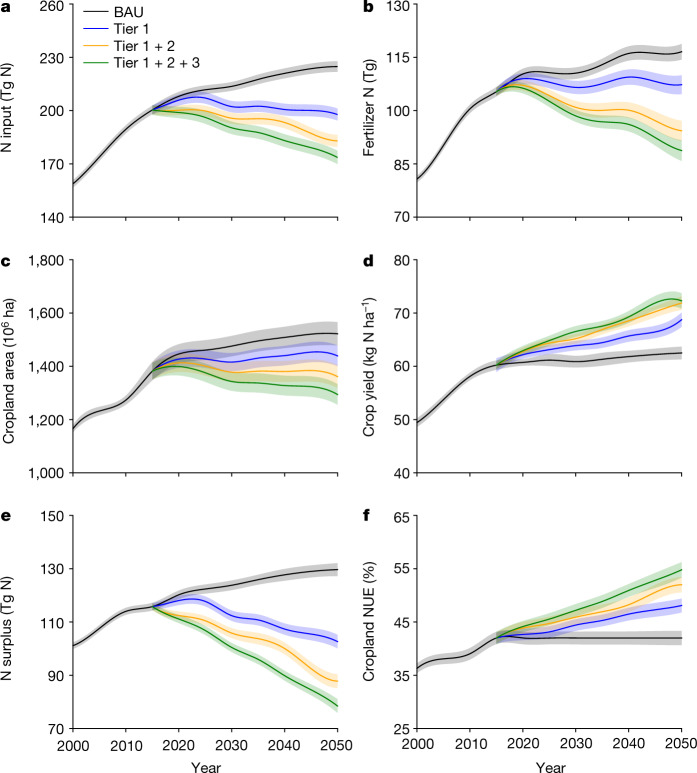
Global cropland N inputs and its uptake and surplus under the tiered approach towards 2050. **a**, Cropland N input. **b**, Fertilizer N use. **c**, Cropping area. **d**, Crop yield. **e**, N surplus. **f**, Cropland NUE. The shadow behind each line shows the 95% confidence interval. Simulated values based on the CHANS model. More details about the scenario settings can be found in Extended Data Table 1. BAU, business as usual. Source data

Therefore, Tier 1 measures have the largest potential to be applied on the global scale and contribute about half of the estimated mitigation potential on N_r_ input and loss to the environment (Fig. 5). Besides a 179 ± 52 billion USD benefit for human health, ecosystems and climate, implementation of Tier 1 measures can bring a 120 ± 29 billion USD benefit resulting from increased crop harvest. The total implementation costs of all Tier 1 measures are negative, at −5 ± 2 billion USD, mainly owing to fertilizer savings and legume rotation that can save fertilization costs (Supplementary Table 2).

We estimated an extra cost of 18 ± 4 billion USD to implement Tier 2 measures globally, which may be a barrier, despite the benefits of 185 ± 55 billion USD, including yield benefits of 104 ± 27 billion USD. The mitigation potential of Tier 2 measures is smaller than that of Tier 1 measures, given its lower implementation potential, especially in less developed countries (Fig. 5e).

Implementation of Tier 3 measures will further improve the cropland NUE and reduce N_r_ losses (Fig. 5). The total net cost for Tier 3 measures was estimated at 25 ± 8 billion USD, although it could save another 7 ± 2 billion USD in fertilizer cost (Fig. 4). Tier 3 measures would require more advanced knowledge and facilities. For instance, modern irrigation systems are self-propelled and equipped with wireless sensors and GPS technology to improve site-specific and volumetric precision of water application to satisfy the needs of the soil and crops. Improving access to information of these measures, for example through farmer-education programmes, can help to create incentives and influence the behaviour of farmers towards more nitrogen-efficient management.

Although the classification of tiers may affect the projection of mitigation potential under different scenarios, it will not change the estimation of the gross cost and benefit if implementing all of these measures. For example, introducing new cultivars is assigned to Tier 3 in this study, as it requires research and development and is possibly expensive at the beginning of adoption. However, once the new cultivars have been widely used in more-developed countries, they can also be more easily adopted by smallholder farmers in less-developed countries, just as with the new cultivars in the Green Revolution in the second half of the twenty-first century.

## Feasibility and application to policy

Mitigation measures for non-point-source pollution have not been widely adopted in many countries owing to various socioeconomic barriers, such as the lack of incentives, insufficient financial assets and knowledge, policy limitations and even social dynamics and cultural concerns^17–19^. Farmers must invest money, labour, knowledge and other essential inputs to achieve these changes, unless there are other drivers, such as agricultural policies with incentives or penalties or other social factors (such as age, gender and education) affecting farmers’ adoption^3^. It is not easy to change farmers’ practices, especially for the large number of smallholders, even with strong evidence of financial gains resulting from practice change^18^. This is largely owing to the low agricultural income ratio (agricultural income divided by the total income) of smallholders^20^. Smallholders normally have part-time jobs in non-agricultural sectors, and the relatively high non-agricultural income suggests an even higher opportunity cost to adopt the abatement measures, as it requires extra labour input from farmers^21^. Although our cost–benefit analysis shows the potential societal benefits of the adoption of these measures, it is an open research question as to how the implementation of these measures can be supported through policies to achieve several wins^19,22^. Policies can promote implementation through various mechanisms, such as restoring price signals by means of internalization of pollution costs^23^, supporting smallholder adoption by granting access to financial capital and by extension services for knowledge transfer^8^, or strengthening non-market social regulating factors^24^.

One suggested policy approach that includes internalization of pollution costs and provides access to financial capital is a NCS that gathers the financial budget from the whole of society who benefit from the N_r_ abatement and food supply^3^. These financial budgets could then be used to subsidize farmers who implement the best management practices for lower pollution and higher yields. Such a NCS could be applied on the national, provincial or local scales if there are clear boundaries within which the implementation costs and benefits can be identified. For instance, if better management practices upstream of a watershed could benefit the water quality of the downstream residents, then the NCS should be applied to the whole watershed scale^25^. For regions in which the boundaries of the impact of the measures are hard to identify, a larger scale could be used to gather financial budgets to cover the implementation costs. The implementation of a NCS would allow farmers to take the social benefits into consideration and mobilize farmers to pursue both yields and societal benefits. The lower limit of the financial budget to subsidize farmers in the NCS is the net implementation costs of all measures (including transition costs such as training and opportunity costs such as non-agricultural income of farmers) within a certain boundary, whereas the upper limit is the total societal benefits (not including yield benefit) of abatement of N_r_ loss. Total societal benefits of N mitigation tend to exceed the costs about 15 times globally; the justifiable NCS financial budget for a region would range between the implementation cost and the total societal benefit^3^ (Fig. 3). In fact, agricultural subsidies are commonly used in many countries to maintain farming profitability that indirectly benefits food security and the environment, and the NCS can be seen as enhancing approaches to better link costs and benefits across society^26^.

In high-income countries, in which access to financial resources is more available owing to existing capital assets and efficient financial markets, environmental pollution could also be internalized by means of a tax on N surplus. This approach aligns with the polluter-pays principle, may circumvent rebound effects (in which efficiency improvements are offset by increased production) and provides incentives for innovation^23,27^. According to this approach, the most effective measures do not need to be preselected by science but can be developed by private-sector agents, which are rewarded on success by lower tax payments^28^. Moreover, tax revenues can be used to forward other policy aims or to balance adverse distributional effects.

Another approach to accelerate better N management is to introduce multi-actor policy schemes bringing together the bearers of the costs and the benefits. An example is the Dutch agri-environment-climate scheme^29^, which tenders collective contracts to local associations that can contain both farmers and civil society actors. This approach may create more innovative and inclusive solutions through social learning. In any case, our study shows that there is a large and yet untapped potential for society to gain from improved policy solutions on agricultural N management. More instruments are also necessary to go beyond the consideration of financial factors. For example, the combination with other instruments or policies, such as biodiversity conservation, may also help to achieve the implementation of measures, especially given the mixed societal benefits of improved N management^30^. Reducing N pollution is an important component of a sustainable transformation of the food system. Beyond mitigating N pollution, such a transformation also requires integration with other societal targets, such as climate change mitigation, biodiversity protection and the wider sustainable development goals.

## Methods

### Data collection and compilation of mitigation measures

We conducted a literature search of peer-reviewed publications after the year 2000 from the Web of Science. For management strategies developed before 2000, we only included those that are still adopted at present. This allowed us to focus on investigating the feasibility of the implementation of the measures that have been applied in recent decades. The keywords used in the search included “N or non-point source pollution”, “mitigation or abatement” and “cropland or farmland”. This paper focuses on the on-farm management practices but excludes the upstream ways of decreasing N pollution, such as diet changes, or decreasing waste beyond the farm, such as manure management in feedlots. Studies were included if they met all of the following criteria: (1) the sample means of the N loss from targeted pathways, yield or NUE were reported for both the control and the treatment groups; (2) details on the experimental location, design and conditions were given to enable cross-checking of duplicate publications; and (3) only field experiments were included.

We included in our analyses a total of 1,521 field observations. We identified and summarized 11 groups of measures, as explained in Supplementary Information 1.1. The treatment groups (and categories) and the control of various management practices were: (1) EEFs (urease inhibitors, nitrification inhibitors, double inhibitors (urease and nitrification inhibitors) and coated or controlled-release fertilizers) were compared with their counterparts without inhibitors or coatings (control); (2) organic amendments (biochar, manure and crop residues) were compared with normal fertilization (control); (3) incorporation of legumes into a rotation was compared with non-legume rotation (control) over the full period of the rotation; (4) wetlands or ponds with a buffer zone were compared with those without a buffer zone on the same study field (control); (5) for optimizing the N fertilizer rate, the highest fertilizer application rate used in the experiment was considered as the control, whereas the other rates were converted to the percentage reduction (<25%, 25–49%, 50–74% and ≥75%) relative to the control; (6) for fertilizer type, urea was treated as the control and the non-urea treatments were categorized into ammonium-based or nitrate-based fertilizers, manure and compost; (7) for fertilizer time, the splitting frequency of fertilizer application was compared with a single application (control); (8) for fertilizer place, deep placement of fertilizers was compared with surface broadcast or topdressing (control), with studies focusing on urea and manure; (9) crop varieties with a high NUE were compared with those with low NUE (control); (10) no-tillage was compared with conventional tillage (control); and (11) drip irrigation or optimal irrigation was compared with normal irrigation (control). All data were extracted from text or tables directly or figures using WebPlotDigitizer 4.2. The full set of publications and outcomes for each is detailed in the Supplementary Information meta-analysis documentation.

### Meta-analysis of mitigation measures

The natural log of the response ratio ($r={\bar{x}}_{{\rm{t}}}/{\bar{x}}_{{\rm{c}}}$, in which ${\bar{x}}_{{\rm{t}}}$ and ${\bar{x}}_{{\rm{c}}}$ are the means of the treatment and control groups, respectively) was used as a metric for the analysis of treatment effects on N_r_ loss from various pathways (NH_3_, NO_*x*_ and N_2_O emission, N runoff to surface water and N leaching to groundwater), yield and NUE. The results were reported as the percentage change under treatment effects ((*r* − 1) × 100). Negative percentage changes indicate a decrease in the variables owing to the management practices, whereas positive changes indicate an increase. We followed a commonly adopted randomization resampling procedure^31^ and generated mean effect sizes and 95% confidence intervals by bootstrapping (4,999 iterations)^32^ using the software MetaWin 2.1 (ref. ^33^). In previous meta-analyses, the effect sizes reported were weighted by the inverse of the pooled variance^34^, replication^35^ or unweighted^36^. The studies in our database did not always include published variances or replications, so the unweighted approach^36^ was adopted in our analysis. The effects of management practices on N_r_ losses, yield and NUE were considered substantial if the confidence intervals did not overlap with zero^31^.

The variations of effects may be due to the changes in local natural conditions, such as soil and climate. These local conditions not only affect the effects of mitigation measures but also the local N cycle. However, given the relatively consistent effect of measures on the N cycle among countries (Supplementary Figs. 4–14 and 17 and Supplementary Table 6), which is indicated by the notable effect of the response ratio of the main N loss pathways and mitigation measures in Fig. 1, we did not categorize the effect sizes based on countries. We thus linked the results of the meta-analysis and national budget modelling through modifying the N cycling parameters in the N budget models using the mitigation potential of each measure in the meta-analysis. Although the average mitigation potential of each measure in percentage change is considered the same in each country, the final mitigation potential is different because the N inputs differ and the implementation of measures varies between nations owing to the heterogeneity of the most appropriate set of practices at a local scale. For the variation in the effectiveness of measures among crop species, may we expect that the largest impact should come from non-legumes relative to legumes, which fix N from the atmosphere. We therefore separated legumes from other crops in the meta-analysis in Fig. 1. In regions with several cropping indexes, such as wheat–maize rotation, it is hard to identify the mitigation effect for each crop type because of the interactions. Thus, we did not consider the effect of other crop species on cropland N use and loss. More details can be found in the following sections.

### Tier classification of mitigation measures

We developed a tiered classification scheme to group similar measures and better facilitate analysis, which is illustrated in Supplementary Fig. 2. The classification criteria mainly focus on four aspects: mitigation efficacy, technical threshold, acceptance by farmers, and the implementation cost. Each measure is thus assigned to one of three tiers as outlined in Supplementary Table 1 based on expert judgement. The three tiers are defined as follows.

#### Tier 1 measures

Measures with low technical thresholds, high mitigation efficacy, low implementation cost and high acceptance by farmers. This includes the use of EEFs, soil amendments and greater legume inclusion in cropland. The use of buffer strips is also classified in Tier 1. Although this measure does not require sophisticated technology and is applicable to both more and less developed countries, it occupies marginal croplands and may threaten food security, reducing its applicability in countries with land scarcity (see Supplementary Table 1). The use of EEFs is a typical Tier 1 measure. Governments can subsidize EEFs to make them the same or lower price compared with traditional fertilizers and farmers would prefer to use these new EEFs given that they can earn more with the same or lower input^37^. Regulation, upscaling and appropriate competition across the industry also hold the potential to reduce EEF prices substantially. A similar tier can also be applied to the use of amendment and legume rotation. Tier 1 can be applied in all global regions, regardless of the farm size or farmers’ knowledge level (Supplementary Table 1).

#### Tier 2 measures

Measures with medium technical thresholds, medium implementation costs and medium acceptance by farmers, which need policy support to promote the application. Measures included here are limited to the use of the right rate, right type, right placement and right time of fertilizer application, namely, the 4R nutrient stewardship. Compared with Tier 1 measures, it is harder for farmers to adopt measures from other tiers (Fig. 4a). Implementation of the 4R measures requires extra inputs from farms, such as knowledge exchange, labour and machinery^10^. Knowledge requires long-term training, which is more attractive to large-scale farmers than smallholders, given the scale effect of fixed inputs, including knowledge, machinery and other fixed assets. Larger farm sizes tend to have lower fixed input per cropland area^38^. Instead of investing time and financial resources in improved agricultural practices, it may often be more attractive for smallholders to invest in off-farm activities, in which they can realize a higher return^39^.

#### Tier 3 measures

Measures with higher technical threshold, higher implantation cost and lower acceptance by farmers owing to trade-offs with other targets (food security, land use, urbanization, land use), which require strong policy intervention and social support to promote their implementation. Measures include increasing adoption of new cultivars with higher yield potential, improved irrigation and no tillage. This tier-based classification may be contrasted with the United Nations Economic Commission for Europe (UNECE) categories 1–3 defined by Bittman et al.^40^, which focus on the robustness of the evidence base for justifying the recommendation of each measure. For example, no tillage is a conservation tillage measure that reduces the compaction of the soil and reduces the water loss by runoff and prevents soil erosion. Although no tillage suggests merely the absence of tillage, several components need to be applied to a conservation agriculture system to guarantee equal or higher yields and better environmental performance than conventional tillage systems. A certain level of experience is needed to establish no-till crops properly and poor crop establishment can arise from both biophysical factors as well as a lack of knowledge by researchers on appropriate equipment, soil conditions, seeding techniques etc.

### Cropland N budget

The CHANS^11^, MAgPIE^12^ and IMAGE^13^ models were used to estimate the global cropland N budget. Details for each of these models can be found in Supplementary Information 1.2. The cropland N budget by country/region in 2015 was established to identify the current cropland N input, output and NUE. N inputs (*N*_input,*i*_) to cropland include five elements, which are N fertilizer input (*N*_fer,*i*_), manure N input (*N*_man,*i*_), biological N fixation (*N*_fix,*i*_), atmospheric N deposition (*N*_dep,*i*_) and irrigation (*N*_irr,*i*_). N outputs from cropland are divided into four elements, which are crop harvest (*N*_harvest,*i*_), N gas emission (*N*_gas,*i*_, including NH_3_, N_2_O, NO_*x*_ and N_2_), N leaching (*N*_leach,*i*_) and N runoff (*N*_runoff,*i*_). On the basis of the integrated model results of CHANS^11^, MAgPIE^12^ and IMAGE^13^, we identified the share of different forms of N loss in different countries/regions. Then the regional N_r_ emission fractions, referring to all N losses including leaching and runoff, were embedded into the CHANS model to perform further mitigation assessment under various scenarios with this model (see Supplementary Fig. 2).

Cropland NUE is defined here as the ratio of harvested crop N to total cropland N input. A target harvest N (*N*_target harvest,*i*_) and NUE (NUE_target,*i*_) was derived, which was assumed to represent a reasonable index of the implementation of current best technologies and management practices. Determination of the regional-specific target NUE_*i*_ for 2050 was based on Zhang et al.^14,41^ and studies that considered the yield potential and environmental boundary^42,43^. These two key indicators allow us to calculate the overuse of anthropogenic N input (Δ*N*_input,*i*_) to the cropland in view of the target harvest N (target NUE) for different countries and regions *i* following equations (1)–(5).

1 $${N}_{{\rm{input}},i}={N}_{{\rm{fer}},i}+{N}_{{\rm{man}},i}+{N}_{{\rm{fix}},i}+{N}_{{\rm{dep}},i}+{N}_{{\rm{irr}},i}$$

2 $${N}_{{\rm{output}},i}={N}_{{\rm{harvest}},i}+{N}_{{\rm{gas}},i}+{N}_{{\rm{leach}},i}+{N}_{{\rm{runoff}},i}$$

3 $${{\rm{NUE}}}_{i}={N}_{{\rm{harvest}},i}/{N}_{{\rm{input}},i}$$

4 $${N}_{{\rm{target}}{\rm{input}},i}={{N}_{{\rm{target}}{\rm{harvest}},i}/{\rm{NUE}}}_{{\rm{target}},i}$$

5 $$\Delta {N}_{{\rm{input}},i}={N}_{{\rm{input}},i}-{N}_{{\rm{target}}{\rm{input}},i}$$

### The reduction potential of N fertilizer use

The estimation of global and regional fertilizer reduction potential was based on the difference between the current and optimal fertilizer use combined with the best cropland N management practices.

6 $$\Delta {N}_{{\rm{fer}},i,k}=\Delta {N}_{{\rm{input}},i,k}+\Delta {N}_{{\rm{man}},i,k}-\Delta {N}_{{\rm{dep}},i,k}$$

in which *k* means the most appropriate set of combinations of several options; we assumed the *N*_fix,*i*,*k*_ and *N*_irr,*i*,*k*_ to change little. Enhanced manure recycling to cropland (Δ*N*_man,*i*,*k*_), which contributes to the reduction in chemical fertilizer input (Δ*N*_fer,*i*,*k*_), has been included in the estimation of future cropland budgets. The baseline manure N input to cropland was derived from the Food and Agriculture Organization of the United Nations (FAO)^44^. The different future estimations were made by adjusting the manure recycling ratio to a value that respects both the regional-specific cropland maximum carrying capacity and the manure production potential. The change of N deposition (Δ*N*_dep,*i*,*k*_) to cropland was assumed to be proportional to the reduction of N_r_ emission. *N*_fer_ cannot be negative and any Δ*N*_fer_ > *N*_fer_ was set to zero.

### Cropland N_r_ mitigation potential

To incorporate the results of the global meta-analysis, the results were first integrated into the three N budget models (CHANS, MAgPIE and IMAGE) using parameterizations (for NUE, yield and emissions). Implementation of these identified measures could change the share of different N use and losses. The calculation of the N_r_ mitigation potential was based on the mitigation efficiency of selected mitigation options for different countries obtained from the meta-analysis and the cropland N mass balance integrated with the N budget models. The percentage change in N losses by the impact of measures is assumed to be equal at the global level but because the N flux differs among countries, the absolute change in N losses varies accordingly. Regional application of the integrated N budget model then allowed the outcomes to be assessed in relation to the national-level budgeting. Regional cropland N mitigation boundaries during the scenario analysis were respected. The detailed process of integration can be found in Supplementary Information 1.3.

The reduction of cropland N loss (Δ*E*_*i*,*j*,*k*_) in the form of NH_3_, NO_*x*_, N_2_O, N leaching and N runoff in country/region *i* was calculated as:

7 $$\Delta {E}_{i,j,k}={A}_{i,j,k}\times [{{\rm{E}}{\rm{F}}}_{i,j}\times {\eta }_{i,j,k}\times {X}_{i,j,k}]$$

in which *j* represents the form of N loss (NH_3_, NO_*x*_, N_2_O emissions, N leaching and/or N runoff) from cropland; *A*_*i*,*j*,*k*_ is the cropland activity data (fertilizer use, cropping area or production); EF_*i*,*j*_ is the corresponding uncontrolled emission factor, for which uncontrolled refers to the baseline model; *η*_*i*,*j*,*k*_ is the specific abatement efficacy; *X*_*i*,*j*,*k*_ is the implementation rate of the abatement technique or options *k*, and the baseline is zero, that is, no implementation of abatement measures.

Moreover, the target NUE and yield in each country were used to constrain the overall mitigation potential when combining different measures because we did not need to apply all of these measures to achieve the target NUE. For measures that do not interact, the mitigation potentials were added (cumulative impacts). For measures that interact, we adopted the results from experiments that combined those measures to estimate their combined mitigation potential (Supplementary Fig. 1).

### Cost–benefit analysis

On the basis of the changes of N_r_ fluxes of national croplands N input and outputs under certain abatement measures, the national N budgets data are used for the cost and benefit calculation. The mitigation costs for N pollution from global croplands in this study are defined as a direct expenditure (the sum of investment costs and operation costs) for the implementation of the 11 measures to reduce N loss from global croplands. Here we mainly refer to the database and methodology of the cost assessment from the online Greenhouse Gas and Air Pollution Interactions and Synergies (GAINS) model^45^ to calculate the global and regional abatement costs. Country-specific agricultural conditions and farming practices have been considered in the GAINS model, including local labour costs, energy prices, farm sizes, costs of by-products and so on. All costs are in constant 2017 USD in this study. A detailed description of the GAINS model and cost calculation can be found in Klimont and Winiwarter^45^. The annual implementation cost (IC_*i*,*k*_) in country/region *i* and N term *k* is calculated as:

8 $${{\rm{I}}{\rm{C}}}_{i,k}=\Delta {E}_{i,k}\times {{\rm{U}}{\rm{C}}}_{i,k}$$

in which UC_*i*,*k*_ represents the integrated unit abatement cost of the most appropriate set of mitigation options to reduce cropland N loss in country/region *i*, which is derived from the online GAINS model database and adjusted according to country-specific farming practices; Δ*E*_*i*,*k*_ is the change in N emission in different forms, such as NH_3_ and N_2_O, which are derived from the integrated modelling analysis in equation (7).

The societal benefits (SOC_benefit,*i*,*k*_) of mitigating N pollution from global croplands in this study is defined as the sum of avoided damage costs of premature mortality by air pollution (HH_benefit,*i*,*k*_), ecosystem health (EH_benefit,*i*,*k*_), yield benefit (YD_benefit,*i*,*k*_) and GHG mitigation benefit (GHG_benefit,*i*,*k*_), as shown in equation (9):

9 $${{\rm{SOC}}}_{{\rm{benefit}},i,k}={{\rm{EH}}}_{{\rm{benefit}},i,k}+{{\rm{HH}}}_{{\rm{benefit}},i,k}+{{\rm{YD}}}_{{\rm{benefit}},i,k}+{{\rm{GHG}}}_{{\rm{benefit}},i,k}$$

Several USA and EU studies have examined the damage cost of N_r_ effect on ecosystems^46–53^. At present, we do not have costs and benefits data available for other nations of the world. For this reason, we assume that the unit N_r_ damage costs (Supplementary Data 1 in the Excel file) to the ecosystems in the EU and the USA are also applicable to other countries after correction for differences in the willingness to pay (WTP) for ecosystem services to assess the benefits and trade-offs associated with N-related management actions for different areas, as shown in equation (10):

10 $${{\rm{E}}{\rm{H}}}_{{\rm{b}}{\rm{e}}{\rm{n}}{\rm{e}}{\rm{f}}{\rm{i}}{\rm{t}},i,k}=\sum _{j}\Delta {E}_{i,j,k}\times {{\rm{\partial }}}_{{\rm{U}}{\rm{S}},j}\times \frac{{{\rm{W}}{\rm{T}}{\rm{P}}}_{i}}{{{\rm{W}}{\rm{T}}{\rm{P}}}_{{\rm{U}}{\rm{S}}}}\times \frac{{{\rm{P}}{\rm{G}}{\rm{D}}{\rm{P}}}_{i}}{{{\rm{P}}{\rm{G}}{\rm{D}}{\rm{P}}}_{{\rm{U}}{\rm{S}}}}$$

in which ∂_US_ is the estimated unit ecosystem damage cost of N_r_ emission in the USA in the 2000s^49,51^; WTP_*i*_ and WTP_US_ are the values of the WTP for ecosystem service in country *i* and the USA, respectively; PGDP_*i*_ and PGDP_US_ stand for the per capita gross domestic product (in constant 2017 USD) of country *i* and the USA, respectively. The welfare implications of transforming damages are based on WTP; the data source of WTP can be found in Supplementary Data 2 in the Excel file.

The health benefit (HH_benefit,*i*,*k*_) refers to the benefit of prevented mortality derived from PM_2.5_ mitigation caused by cropland N_r_ abatement^15^. We derived the national-specific unit health damage costs of N_r_ emission from the methodology of Gu et al.^15^, which connected the economic cost of mortality per unit of N_r_ emission with the population density, gross domestic product per capita, urbanization and N-share. The calculation of health benefits from cropland N management is shown in equation (11):

11 $${{\rm{H}}{\rm{H}}}_{{\rm{b}}{\rm{e}}{\rm{n}}{\rm{e}}{\rm{f}}{\rm{i}}{\rm{t}},i,k}=\sum _{j}\Delta {E}_{i,j,k}\times {{\rm{H}}{\rm{C}}{\rm{o}}{\rm{s}}{\rm{t}}}_{i,j}$$

in which Δ*E*_*i*,*j*_ is the estimated reduction in cropland N_r_ emissions and HCost_*i*,*j*_ represents the unit health damage cost of N_r_ emissions (values can be found in Supplementary Data 2 in the Excel file).

The yield benefit (YD_benefit,*i*,*k*_) refers to the extra economic benefits from increased crop yield, as shown in equation (12):

12 $${{\rm{Y}}{\rm{D}}}_{{\rm{b}}{\rm{e}}{\rm{n}}{\rm{e}}{\rm{f}}{\rm{i}}{\rm{t}},i,k}=\Delta {H\nu }_{i,k}\times {{\rm{Y}}{\rm{P}}}_{i,k}$$

in which Δ*Hν*_*i*,*k*_ is the change in total harvest N from cropland and YP_*i*,*k*_ is the integrated crop price in USD per kg N calculated on the basis of the FAOSTAT database. The national specific value can be found in Supplementary Data 2 in the Excel file.

For monetary evaluation of the climate impact, we used the regional-weighted N_r_ damage cost to multiply with the reduction of N_r_ emission, as shown in equation (13):

13 $${{\rm{G}}{\rm{H}}{\rm{G}}}_{{\rm{b}}{\rm{e}}{\rm{n}}{\rm{e}}{\rm{f}}{\rm{i}}{\rm{t}},i,k}=\sum _{j}\Delta {E}_{i,j,k}\times {{\rm{C}}{\rm{C}}{\rm{o}}{\rm{s}}{\rm{t}}}_{i,j}$$

in which CCost_*i*,*j*_ represents the unit abatement cost to the climate in USD per kg N (values can be found in Supplementary Data 2 in the Excel file). We account for the effects in which N_2_O contributes to global warming, whereas NO_*x*_ and NH_3_ emissions have a cooling effect on the global climate^54^.

### Future scenario setting under the tiered approach

The business-as-usual scenario is a baseline scenario and is assumed to maintain current farming practices, with no further improvement of cropland N management. Another three scenarios were assigned with three different packages of measures (Extended Data Table 1). The classification criteria of tiered measures mainly focuses on four aspects: mitigation efficacy, technical threshold, acceptance by farmers and implementation cost.

Incentivizing the implementation is complicated, which is also the reason why many measures developed in the past decades are still not being implemented in many regions/countries. An example is the use of soil testing to rationalize N fertilizer rates and soil management. After confirming the mitigation effects, local socioeconomic and natural conditions generally determine whether these measures should and can be implemented. For example, EEFs should be acceptable for Chinese smallholders if the government subsidizes these fertilizers to have the same or even lower price compared with conventional fertilizers. However, the 4R nutrient stewardship would not be easily applied for smallholders in China and elsewhere, given the required knowledge transfer and machinery for the implementation. Therefore, we assume that different measures could be applied in different countries based on their socioeconomic and natural conditions, which are the key criteria of our tiered approach.

Generally, different tiers are suitable for different regions and countries. We used targeted NUE as the final criterion to assess whether we need to implement more measures to achieve the goal. The implementation ratio of different measures in different countries is estimated on the basis of their socioeconomic and natural conditions. For instance, some countries reported that they have a low implementation rate of EEFs owing to insufficient financial support. Then we would judge whether the NCS could support such an implementation for the targeted NUE. In African countries, the lack of access to N fertilizers has caused soil N depletion and yield reduction. Thus, other than mitigation measures, we recommend more fertilizer use in these countries. This would slightly increase the N loss within the allowable N-carrying capacity in the planetary boundary.

For each measure, we estimate different adoption rates and mitigation efficiencies by country on the basis of their croplands’ N budgets, crop yield, farm size and management practices, as summarized in Supplementary Table 5. Human population (population density) and economic level (per capita gross domestic product) are two key determinants of future food demand and required N_r_ harvest from cropland, which are assumed to be consistent under all tiered scenarios. It is assumed that the adoption of measures will change the cropland N input, NUE, crop yield and cropping area, to maintain the required N_r_ harvest for human consumption. Details on data sources, prediction methods and parameters can be found in Supplementary Information 1.

### Reporting summary

Further information on research design is available in the Nature Portfolio Reporting Summary linked to this article.

## Online content

Any methods, additional references, Nature Portfolio reporting summaries, source data, extended data, supplementary information, acknowledgements, peer review information; details of author contributions and competing interests; and statements of data and code availability are available at 10.1038/s41586-022-05481-8.

### Supplementary information

Supplementary Information This file contains Supplementary Methods; Supplementary Discussion; Supplementary Tables; Supplementary Figures and Supplementary References.

Reporting Summary

Supplementary Data 1

### Source data

Source Data Fig. 1

Source Data Fig. 2

Source Data Fig. 3

Source Data Fig. 4

Source Data Fig. 5

Source Data Extended Data Fig. 1

Source Data Extended Data Fig. 2

Source Data Extended Data Fig. 3

Source Data Extended Data Fig. 4

Source Data Extended Data Fig. 5

Source Data Extended Data Fig. 6

## Figures and Tables

**Extended Data Table 1:**
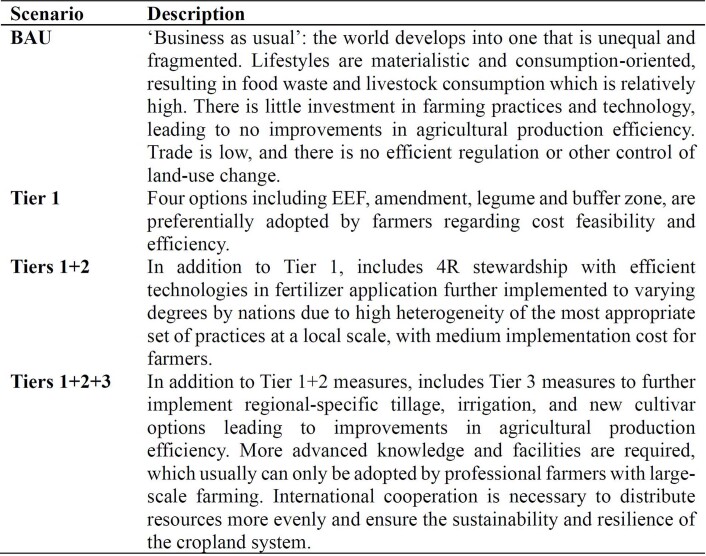
Scenario setting and characteristics

4R refers to the combined options of the right fertilizer type, right amount, right placement and right time of fertilizer application. More detailed descriptions of the scenario settings and parameterization can be found in the Supplementary Information text.

## Data Availability

The literature used in the meta-analysis is listed in Supplementary Data 10 in the Excel file. A more detailed methodology can be found in Supplementary Information 1. Extended data of the main findings and further discussion can be found in Supplementary Information 2. Source data are provided with this paper.
