# Publisher Correction: Cost-effective mitigation of nitrogen pollution from global croplands

**DOI:** 10.1038/s41586-023-05708-2

**Published:** 2023-01-16

**Authors:** Baojing Gu, Xiuming Zhang, Shu Kee Lam, Yingliang Yu, Hans J. M. van Grinsven, Shaohui Zhang, Xiaoxi Wang, Benjamin Leon Bodirsky, Sitong Wang, Jiakun Duan, Chenchen Ren, Lex Bouwman, Wim de Vries, Jianming Xu, Mark A. Sutton, Deli Chen

**Affiliations:** 1 College of Environmental and Resource Sciences, Zhejiang University, Hangzhou, China; 2 Policy Simulation Laboratory, Zhejiang University, Hangzhou, China; 3 School of Agriculture and Food, The University of Melbourne, Melbourne, Victoria Australia; 4 Key Laboratory of Agricultural Environment of the Lower Reaches of the Yangtze River, Institute of Agricultural Resources and Environment, Jiangsu Academy of Agricultural Sciences, Nanjing, China; 5 PBL Netherlands Environmental Assessment Agency, The Hague, The Netherlands; 6 School of Economics and Management, Beihang University, Beijing, China; 7 International Institute for Applied Systems Analysis, Laxenburg, Austria; 8 China Academy for Rural Development, Zhejiang University, Hangzhou, China; 9 Department of Agricultural Economics and Management, School of Public Affairs, Zhejiang University, Hangzhou, China; 10 Potsdam Institute for Climate Impact Research (PIK), Potsdam, Germany; 11 Department of Earth Sciences - Geochemistry, Faculty of Geosciences, Utrecht University, Utrecht, The Netherlands; 12 Environmental Systems Analysis Group, Wageningen University & Research, Wageningen, The Netherlands; 13 Zhejiang Provincial Key Laboratory of Agricultural Resources and Environment, Zhejiang University, Hangzhou, China; 14 Edinburgh Research Station, UK Centre for Ecology & Hydrology, Penicuik, UK

**Keywords:** Element cycles, Agriculture

Correction to: *Nature* 10.1038/s41586-022-05481-8 Published online 4 January 2023

This paper was originally published under a standard Springer Nature license (© The Author(s), under exclusive licence to Springer Nature Limited). It is now available as an Open Access paper under a Creative Commons Attribution 4.0 International license, © The Author(s). Additionally, the maps in Figure 3d,e were mistakenly reversed (e.g., Alaska should now appear in red in the top-left of Fig. 3d), while in Figure 5c, the y-axis label for cropland area read “10^3^ ha” rather than “10^6^ ha” as corrected now. The errors have been corrected in the HTML and PDF versions of the article and the source data for are updated.

