# Supplementary material for: Cost-effective mitigation of nitrogen pollution from global croplands
Source: Nature. 2023 Jan 4;613(7942):77–84. doi: 10.1038/s41586-022-05481-8 (PMC9842502; doi:10.1038/s41586-022-05481-8)
Supplement: Supplementary file 1 — This file contains Supplementary Methods; Supplementary Discussion; Supplementary Tables; Supplementary Figures and Supplementary References. [file 41586_2022_5481_MOESM1_ESM.pdf]

nature portfolio

https://doi.org/10.1038/s41586-022-05481-8

Supplementary information

Cost-effective mitigation of nitrogen pollution from global croplands

In the format provided by the authors and unedited

nature portfolio

https://doi.org/10.1038/s41586-022-05481-8

Supplementary information

Cost-effective mitigation of nitrogen pollution from global croplands

In the format provided by the
authors and unedited

Supplementary Information for:

Cost-effective mitigation of nitrogen pollution from global croplands

Contents

S1. Supplemental Methods 2

S1.1 Criteria for selection and description of effective N mitigation options for meta-analysis 2

S1.2 Multi-model integration to deliver plausible cropland N budget.. 3

S1.3 Integration of meta-studies into CHANS model.. 5

S1.4 Scenario setting and simulation 5

S2 Supplementary Discussion 6

S2.1 Uncertainty analysis.. 6

S2.2 Limitations 8

S2.3 Challenges.. 10

S3 Supplementary Tables.. 12

Table S1 Detailed description of the selected mitigation options for the Tiered approach 12

Table S2 Description of the way implementation cost and benefits of the different mitigation measures are calculated and their barriers for application................ 14

Table S3 Data sources used for assessing global and regional cropland N budgets and mitigation efficiency with assumptions about data variation and type of uncertainty 20

Table S4 Uncertainty analysis 22

Table S5 Determination criteria of the potential implementation rate for different tiered mitigation measures in the tiered scenario setting 25

Table S6 Correlation coefficient of impacts of mitigation measures on different N use/loss in different countries.. 27

Supplementary Figures.. 30

Figure S1 | Compilation of global cropland N budgets based on multi-model combination 30

Figure S2 | Classification criteria for three tiers of mitigation measures adopted in this study.. 31

Figure S3 | Integration of meta-analysis with CHANS model to perform scenario analysis 32

Figure S4 | Effects of enhanced efficiency fertilizers on N_r loss from croplands. 33
Figure S5 | Effects of organic amendment on N_r loss from croplands................. 34
Figure S6 | Effects of legume rotation on N_r loss from croplands........................ 35
Figure S7 | Effects of the buffer zone on N_r loss from croplands......................... 35
Figure S8 | Effects of right rate of N fertilizer application (reduction) on N_r loss from croplands.. 36
Figure S9 | Effects of the fertilizer type on N_r loss from croplands 37
Figure S10 | Effects of the N fertilizer time in terms of split application of N fertilizers on N_r loss from croplands. 38
Figure S11 | Effects of N fertilizer place, in terms of deep placement on N_r loss from croplands.. 39
Figure S12 | Effects of high NUE cultivar on Nr loss from croplands................. 39
Figure S13 | Effects of reduced irrigation or drip irrigation on N_r loss from croplands. 40
Figure S14 | Effects of no-tillage on N_r loss from croplands................................. 41
Figure S15 | Steps of uncertainty analysis using Monte Carlo simulation........... 42
Figure S16 | Uncertainty contribution of cropland N budgets by regions 43
Figure S17 | Impacts N mitigation measures on N use/loss across different countries 44
References:.. 45

S1. Supplemental Methods

S1.1 Criteria for selection and description of effective N mitigation options for meta-analysis

Four aspects were considered for meta-analysis while choosing abatement measures to the reduce Nr losses to (NH_3, NO_x, N_2O emissions) and water (N leaching and runoff):

1) **Synergies and no or limited trade-offs**: measures were prioritized that can simultaneously reduce the total Nr (NH_3, NO_x, N_2O, N leaching, and runoff) emissions while increasing crop yield are included, such as the use of enhanced efficiency fertilizers (EEFs) which could reduce total Nr emission by 15-40%, while increasing crop yield by 9.1-20.5%; measures that e.g. reduced NH_3 emissions but enhanced N_2O emissions or N leaching, were selected with caution to avoid trade-off.

2) **High mitigation efficiency**: measures that could significantly reduce one or more of the Nr losses are considered, such as deep manure placement, which has a high mitigation potential of 93-99% for NH_3 emissions with limited trade-off to other emissions.

3) **Low implementation cost**: measures with lower costs or labor inputs that farmers prefer, such as tillage and buffer zone are included.

4) **Practical applicability**: measures with limited applicability due to technical, political, or

obvious social barriers were excluded. For example, soil testing was ruled out in this study due to high costs for small farms and high spatial and temporal variability.

A total of 11 mitigation options for specific cropping systems were included in this study for achieving both agronomic and environmental targets based on the selection criteria. Detailed descriptions of their applicability and implementation costs are listed in **Table S1** and **Table S2**. It is noted that there are other measures available in some parts of the cropping systems, e.g., manure management, which offers further opportunity to reduce wasted N, when considering the interaction of livestock and cropping systems, in addition to issues about consumption change. But these measures are mainly not implemented on croplands, thus, we did include them in this study.

As can be seen from the meta-analysis of experimental studies an individual measure may reduce one form of N pollution, while simultaneously increasing another form of N pollution. This may happen, for example, the incorporation of fertilizer or manure into soil reduces volatilization losses, leaving more N remaining in the soil, which is either available for plant uptake or increased leaching. The example also illustrates the potential for interactions between measures.

These potential interactions between measures and above-mentioned compensatory actions are out of the scope of this study, but have been discussed in detail by the UNECE[1] who have identified 24 principles for integrated sustainable N management in agriculture, where the application of these principles allows the development of coherent packages of measures. For example, according to the UNECE[1] Principle 6, to fully realize the benefit of N savings through mitigation, N inputs need to be reduced or harvested produced increased. Hence reducing N losses may allow reduction of N inputs as a compensatory action, thereby saving money and increasing system resilience. The application of these principles will in general allow improved agricultural performance in developing packages of measures. Since it is not possible to treat such interactions fully in a global modeling framework as outlined here, the results may be considered as conservative concerning the estimated benefits.

S1.2 Multi-model integration to deliver plausible cropland N budget

CHANS, IMAGE, MAgPIE model are three main N flow and flux models that could provide croplands N budgets. However, there are great differences among the three models in terms of model principle, targets, process, and data source. Here we integrated the three models by comparing the model outputs. The Nr emission-related parameters (emission fraction and emission factors) were extracted from the three models to get the average-weighted values, which were then reimported into the CHANS model to optimize the parameters of the CHANS model (Figure S1). The multi-model integration provides the new adjusted cropland N budgets and N_r emission inventories by nation. For future scenario simulation and analysis, the meta-analysis results were integrated into the optimized CHANS model (Figure S3) to assess the potential, cost, and benefits under assumed scenarios.

It is noted that N_2 emission was estimated based on the mass balance of N cycling in the cropland system. The absence of N_2 data in the meta-analysis is mainly due to there being no experimental studies reviewed to show the effects of measures on N_2; however, it can be estimated on the cropland system level to quantify the N savings.

The CHANS model includes baseline estimates for present conditions of manure production and of its fate including wasteful disposal or effective recycling in agriculture [2, 3]. For the future scenario setting a modification of this approach was needed where we estimated the animal population and manure production consistent with the demand of human consumption. We first predicted the required livestock population to meet the human consumption, and then referred to the FAOSTAT and recent studies that suggest the cropland carrying capacity and environmental boundary to determine the recycling ratio, finally calculated the amount of manure N returned to the cropland. The potential of manure N being effectively recycled is related to the meta-analysis of field experiments. Composting and fermentation of organic waste are counted as organic recycled manure and are used in cropland as a soil amendment in this study.

Below is a brief description of the three models. These three N budget models are widely used globally, thus, we did not list all the details of these models. For further data source and model simulation processes, please read the original studies about these three models.

CHANS model. CHANS (Coupled Human And Natural Systems) is a N-flow process-based model that accounts for the complex biogeochemical processes of N cycling[2, 3]. The CHANS-Globe integrates the fluxes of N input, output, and accumulation among 14 subsystems (cropland, feedlot, grassland, human, industry, aquaculture, forest, pets, urban lawn, solid waste, wastewater, atmosphere, surface water and groundwater) based on the embedded mass balance approach. N fluxes regarding cropland subsystems are extracted to compile the Cropland N budget. The main data sources for CHANS-Globe are (i) the global statistics databases[4-7] that provide social-economic and agricultural activity data at the national or regional scale, including human population, GDP, land use, crop/livestock production, farm productivity, N fertilizer use, etc.; (ii) latest published field experiments, literature studies, and national pollutant inventories[8-12] that offer diverse parameters (e.g., N content in crops, harvest index, manure recycling ratio) and national-specific N_r emission factors (EFs). A detailed description of the CHANS model framework and calculation could be found in Gu et al. (2015, 2020)[2, 3].

IMAGE model. IMAGE (Integrated Model to Assess the Global Environment) is an integrated assessment model that simulates the environmental consequences of human activities worldwide[13]. It represents interactions between society, the biosphere, and the climate system to assess sustainability issues such as climate change, biodiversity and human well-being. The objective of the IMAGE model is to explore the long-term dynamics and impacts of global

changes that result from interacting socio-economic and environmental factors[13]. For a more detailed introduction on the IMAGE model: see the IMAGE website (https://models.pbl.nl/image/index.php/IMAGE_framework).

MAgPIE model. MAgPIE (Model of Agricultural Production and its Impact on the Environment) is a global land-use allocation model with a spatial resolution of 0.5°x0.5° that is linked to the grid-based dynamic vegetation model Lund-Potsdam-Jena managed land model (LPJmL)[14]. The data preparation routines MADRaT (May All Data be Reproducible and Transparent) and mrcommons (MadRat commons Input Data Library)[15] for MAgPIE use a N budget approach[16] in combination with historical FAOSTAT data to drive a comprehensive coverage of N cycle and flows.

S1.3 Integration of meta-studies into CHANS model

The literature review and meta-analysis of many field experiments, i.e. 1521 field observations in the past two decades, of eleven key measures that can mitigate N pollution from croplands was used to provide global and regional N mitigation changes for the period 2020-2050 (Figure S2). The results for the specific mitigation measures are summarized in Figures S4-S14. All the mitigation options have limited mitigation efficiency and always come with an uncertainty range due to the changes in local natural conditions such as soil and climate[17], and in this study, the uncertainty ranges of mitigation options are shown in Figures S4-S14 alongside the mean value.

In this study, we first evaluate the baseline global cropland-N_r emissions in 2015 using the process-based CHANS model that can be applied across locations and regions with N-rate-dependent emission factors (EFs), as specified in the Supplementary data S2. Then, the global cropland meta-analysis results are integrated into the CHANS with the proposed tiered measures at a national scale based on their current farming practice, N application rate, and their social-economic level (Figure S2). The quantitative mitigation impact of screened tier measures on N_r loss, crop yield, and cropland NUE were scaled and fitted by the regional N input intensity, yield gap, and NUE to be integrated into the N budget calculation. The integration of meta-analysis and CHANS model simulation provided a new benchmark for cropland N management and is expected to inform advancement towards more realistic and effective mitigation pathways via the tier measures.

S1.4 Scenario setting and simulation

To explore the mitigation potential and strategy of cropland N management, we apply a baseline scenario following Shared Socio-Economic Pathway 5 (SSP5)[18, 19]. The baseline scenario is characterized by rapid and fossil-fueled development with high socio-economic challenges to mitigation and low socio-economic challenges to adaptation[19]. This scenario follows socio-economic development prediction as a business-as-usual (BAU) scenario and provides a

complete and consistent dataset on levels of human consumption, technical development, and environmental awareness for future scenario analyses. The basic elements (population, GDP) are summarized in Supplementary data S5. The regional Cropland NUEs for the year 2020-2050 are assumed to be constant with the year 2015 in the BAU scenario.

Against this BAU scenario, three-tiered scenarios were integrated into the CHANS model corresponding to packages of mitigation measures as follows: tier 1, tier 1+2, and tier 1+2+3 measures, with the scenarios (Tier 1 Scenario, Tier 1+2 Scenario, Tier 1+2+3 Scenario) named according to these measure packages. Detailed data collection and compilation for each scenario are summarized in Supplementary data S5-S9. Then the assumed scenario was integrated into the CHANS model to quantify future cropland N budgets, including N stocks, flows, and transformation, and identify the reduction potential for N losses by nation.

The criteria to determine the implementation rate of specific tiered mitigation measures are summarized in Table S5. Generally, it assumed different measures with varying implementation rates in different regions based on their current farming practices and social-economic level.

The cropland N budget at the national scale under a BAU scenario is compiled firstly assuming that the harvest N from global croplands could meet the demands of human consumption on crop food and animal food, then the improved farming practices under assumed tier scenarios will be expected to have the same amount of harvest N but with varied NUE, crop yield and cropping area, which is set according to meta-analysis and mass balance principle (Detail data compilation could be found in Supplementary data S5-S9). Then, the future cropland N budgets, including N surplus, N_r loss will be calculated in the optimized CHANS model.

S2 Supplementary Discussion

S2.1 Uncertainty analysis

We made an uncertainty analysis of the cropland N budget and the cost-benefits under assumed tier scenarios using the Monte Carlo simulation. Monte Carlo simulation generates a set of random numbers according to the data distribution and parameters for each variable. In this study, the Monte Carlo simulation is integrated into the optimized CHANS with 1000 simulations (Figure S15). Data collected from global and regional statistics, surveys, and other sources provided the average values, while their relative uncertainty (given by the coefficients of variation, CV) was estimated based on published literature and authors' expert judgment. Variables and parameters obtained from international publications were ascribed to high CVs. A set of random numbers are then generated to the specific inputs, including activity data, parameters, and emission factors according to their uncertainty level and distribution type. After generated, all sets of variable values are input into the CHANS model to compile croplands N budgets.

The total variance of a N budget is equal to the sum of the variances of the various flows plus twice the covariance of all possible two-way combinations of these flows. We identified the uncertainties for various N flows and their contributions to total uncertainties (Table S4). The regional uncertainties in cropland N budgets are illustrated in Figure S16.

The average uncertainty of the cropland budgets was calculated at 19%. The cropland N budget inputs term with the largest contribution to uncertainty in the estimation of fertilizer N (32%) and BNF N (36%). Uncertainties in synthetic N fertilizer use are mainly due to a lack of recorded information on ratios of synthetic and recycled nutrients in compound fertilizers. Fertilizer input data collected from the FAOSTAT did not provide high-resolution information on fertilizer types and application methods. Only a few estimates of BNF rate on regional scales are available for the CHANS model to refer to, we, therefore, use the average values for all the nations, which induced large uncertainties. The uncertainties of the social costs and benefits of optimized 2015 were estimated at 25% and 24%. The estimation of climate impact has the highest uncertainty at 50% but low uncertainty contribution to total benefits mainly due to the Nr damage cost to the climate is still not well studied. The range uncertainties of the global cropland N inputs are dominated by China, India, and the Other Asian counties (Figure S16.a), which together account for 56–64% of the combined global N inputs and outputs in 2015.

Uncertainty analysis shows that the variation in data sources, spatial heterogeneity in cropland activities and insufficient knowledge of N cycling, and imperfect model parameterization are the three dominant sources of uncertainties. Firstly, the variation in data sources implies high uncertainty. The scarcity of fine data on the spatial and temporal distribution of N inputs and outputs, for example, the application of synthetic fertilizers and animal manure by the crop may let out the accuracy of the regional N budgets. The further assessment of mitigation costs and benefits for humans and the ecosystem is expected to have a larger uncertainty due to the amplification effects of the uncertainty in the related calculation process.

Secondly, this study builds on established models and methods but goes beyond previous research by combining data from FAOSTAT, meta-analysis, and a range of national data sources. This methodology has inevitably simplified the complex N cycling and left out spatial variation. Considering the insufficient understanding and involvement of spatiotemporal heterogeneity in biogeochemical and hydrological processes, using this national-level N budget to explore trajectories of N use and evaluate their environmental impact may lead to biases and uncertainties. However, these uncertainties are systematic rather than random, and therefore do not affect conclusions based on spatial and temporal comparisons. Future advancement of knowledge and methodology in N cycling and input-output relations could help improve the model performance.

Thirdly, such global assessment as conducted in this study is always limited by the lack of

studies in some world regions, such as south America, Africa, eastern Europe and some parts of southeast Asia. However, the most data poor regions tend to be those with higher NUE, associated with lower levels of N input. This means that few measures are needed in these regions to apply to achieve the target NUE. In Africa, we even needed to simulate using more fertilizer to increase yield while slightly decreasing the NUE there, in order to avoid soil N mining. For this reason, Fig. 2-4 in the main text show few changes on the N loss and cost-benefit of N mitigations in these regions. In principle, measures could still be applicable in these regions to reduce losses of limited amounts of available Nr, as an alternative to increasing fertilizer or other Nr inputs. Further examination of the relative merits of such alternatives is a matter for future analysis. According to the methodology of the present study, the major nations that applied the measures as described come from the regions that have more scientific studies including China, India, west Europe, and North America.

S2.2 Limitations

The limitations of this study are mainly related to issues with the sample size of meta-analysis and methodological selection.

Meta-analysis. In this study, we combined the N_r mitigation efficacy conclusions from a global meta-analysis with the CHANS model to conduct future scenario analysis. Meta-analysis is a very useful tool for combining the results of studies to boost the precision of our conclusions. However, due to very few studies in central and south America, Africa, eastern Europe, and some parts of southeast Asia, some management practices simply haven't been tested in some regions. Nevertheless, robustness in our approach is shown by the broadly consistent performance of mitigation approaches across a wide range of conditions, as shown by Figure S17 and Table S6. According to this approach, we recognize that total emissions vary substantially, as recognized by variation in activity statistics, while mitigation effectiveness tends to be more conservative. Nevertheless, there are also social differences across contexts, which may affect mitigation potential. Although our translation of the measures for upscaling using the tiered approach provides a broad consideration of such social and economic differences, it is evident that such a global approach necessarily includes simplifications that cannot perfectly represent reality.

Model integration. In this study, we used multi-models to coordinate all the structural parts to perform a comprehensive assessment of cropland N management: data collection, meta-analysis, N cycling model integration, scenario simulation, and cost-benefits analysis. Although multiple methods may be used to their best advantage, unintended consequences may occur due to mismatch or mis-link data and analysis. The following specific issues may be highlighted: a) simplification due to combination of data on different spatial scales, b) simplification due to overall averaging, where some parameters may be non-linear; c) approximation and limitation necessary to keep models sufficiently simple to allow integration of results at a global / regional

scale, leading to some issues being neglected (e.g. process simplification, difficulty to express a comprehensive suite of social interactions in quantitative terms). Here our interdisciplinary team members opt for a pragmatic approach that works closely to link different methods and data and spark ideas across methodological boundaries, while recognizing that such a global approach necessarily requires such simplifications.

There is a huge climate dependence on the percentage of N excreted by animals that is volatilized as NH_3, which is not treated by any of the mainstream globally mapped emissions inventories at present. This does not mean we reject all global emission inventories; rather we take note of the limitations, and indicate uncertainties as far as possible. For the regional Nr effects, we have used local scaled parameters or indexes to estimate the mitigation potential, cost, and benefits. In applying the combined modelling system, the tiered measures from the NCS system are taken using the different combinations at various levels that suit the local conditions.

Cost-benefit analysis. Cost-benefit analysis (CBA) in this study can examine the cost-effectiveness of different mitigation alternatives to balance the productivity and cost-benefits with human health and ecosystem considerations, but this approach has its limitations. The annual costs scaled to different regions were based on agricultural labor wages and input costs such as fertilizers and irrigation. This highlights the importance of considering the likelihood of events over time and the importance of focusing on long-term horizons when evaluating cropland N mitigation and adaptation policies. However, the application of CBA without considering the economic efficiency of the target level cannot deal with the uncertainties associated with cost estimates for ecosystem and climate change impacts. While recognizing these limitations, the estimated uncertainty limits (Table S4) in the CBA indicate that the adoption of selected mitigation options would generate significant net social benefits, estimated annually as 514 [435-567] billion USD.

Meanwhile, it is difficult to accurately quantify the implementation cost and benefit of N pollution reduction on a small scale such as the watershed scale. Compared with the climate effect of carbon on the global scale, the environmental and health effects of N are more local and regionally relevant. For example, the NH_3 emission from N fertilizer use can contribute to the $PM_{2.5}$ pollution on a regional scale downwind, while biodiversity impacts can be local. Therefore, although it is not exactly trading credits among farmers who implemented the measures, and people who benefit from such a N reduction, on a national scale it should be approximately balanced between costs and benefits. At the same time, it is also possible to apply the NCS on the provincial or county scale if clear boundaries of implementation cost and health and environmental benefits could be identified.

Effects of N occur on multiple spatial scales and assessment needs to recognize environmental

climatic, economic and social factors, which also affect policy solutions. In practice for any study, it is only possible to consider such factors partially. This does not mean that all such regional or global assessments are irrelevant. Rather, it means that the limitations need to be transparently acknowledged, and as far as possible the consequent uncertainties quantified or qualitative caveats given. This point may be very simply illustrated with reference to the climate dependence of N emissions to the atmosphere.

Social factors. Many factors affect whether a farmer will or will not adopt a certain technology or improved practices**.** Except for agricultural policy and incentives, previous studies have concluded that many other social factors, e.g., age, gender education, farm experience influence the adoption of advanced technology or methods to improve cropland productivity and sustainability[20-22]. Age was negatively related to technology adoption mainly because older farmers are more experienced, and therefore able to discern the importance of improved technology more as compared to the less experienced young farmers. Besides, older farmers are more likely to have more sources of income compared with their younger counterparts. Male farmers are likely to have more access to inputs, capital, and information through farmers' networks and contact with extension agents than female farmers. Farmers with a low level of education may be hesitant to test innovations, which may hinder technology adoption. Future research may incorporate social norms and risks into decision-making processes, theoretically and empirically. Direct evaluation of the effects of social factors is required; experiments or surveys, as well as the inclusion of expertise from other domains, may assist in this endeavor[22].

The results obtained can also help cropland management stakeholders to implement the tiered measures and to evaluate the suitability of those measures across the diverse range of agricultural management practices. Nevertheless, the uncertainties associated with the method should be reduced by complementing other assessment methods, including more detailed cropland N source assessment, in situ measurements, and information and process modeling.

Having accounted for macroeconomic differences, we agree that there may also be other cultural differences which it is not feasible to account for, such as relative regional importance assigned to different threats (e.g atmospheric visibility is apparently be rated more important in North America than in Europe). It is only possible to recognize transparently the limitations of the methodology and available data, which may highlight future research needs.

S2.3 Challenges

The approach presented in this study is a systematic and flexible scenario framework for building long-term global and national cropland N mitigation scenarios by varying 2050 emissions. The Tier strategy could be a useful tool for reducing N_r emissions that harm human and environmental health. However, potential social and political issues must also be considered. To begin with, there are significant N disparities between different places on the

earth. Applying the present modeling approach would have the potential to create unequally dispersed risks and advantages. That is why it is critical that research into the societal and ecosystem ramifications, as well as investment in the technologies themselves, be adequately financed.

Second, the biggest impediments to improved agricultural N management are capital costs, operational costs, and end-user neglect[23]. In N-limited regions such as Africa and large areas of Latin America, there is often a lack of resources and financial support. This can limit the action to the application of measures that have low capital expenditure requirements and can quickly justify themselves financially, for example, based on the value of reducing total amounts of N_r wasted, allowing improved productivity.

Third, is the political difficulty of enacting mitigation programs in the face of risk and uncertainty. This uncertainty can often lead to a lower degree of priority being assigned, especially in political decision-making processes related to prevention and mitigation investments. As a result, any NCS or similar policy approach should be established and implemented according to a well-organized and successful plan. At the same time actions may be encouraged that improve the confidence in the use of the mitigation approaches, e.g., not just reduced emissions, but demonstrating consistency in improved harvests and increased resilience / reduced vulnerability in the face of fertilizer price rises. However, a concrete implementation plan for accomplishing such a goal has yet to be defined and assessed at the farm level, including consideration of wider landscape, and regional and transboundary interactions.

S3 Supplementary Tables

Table S1 Detailed description of the selected mitigation options for the Tiered approach

Tier	Measures	Description
1	EEF	Purchase and application of enhanced efficiency fertilizers (EEFs), which we define here to include slow/controlled-release fertilizers and nitrification and urease inhibitors
1	Amendment	Use of soil amendments generally is a cost-effective practice. A variety of products are available in bagged or bulk form. Organic amendments include sphagnum peat, wood chips, grass clippings, straw, compost, manure, biosolids, sawdust, and wood ash. Manure-based compost and plant-based composts may be applied at higher application rates, more effectively improving the soil.
1	Legume	Legume–cereal rotation is the practice of using legumes to provide complementary N in the soil for the succeeding crops.
1	Buffer Zone	Applying a buffer zone, being an area located between a certified production operation or portion of a production operation and an adjacent land area that is not maintained under organic management.
2	Rate	Optimizing N fertilizer rate based on soil N supply and crop needs. The optimal N application rate for each nation is estimated based on meta-analysis results.
2	Type	Shifting from urea to ammonium sulfate/nitrate accompanied by lime
2	Time	Splitting fertilizer application into smaller applications throughout the growing season that coincide with the times that the crops most need fertilizer.
2	Placement	The minimum depth of the deep placement of fertilizer N is set at 5 cm below the soil surface, usually supplied at 20cm depth from the soil surface below plants.
3	New Cultivar	Adoption of improved crop varieties is an important agronomic tool. Cultivars not only need to be adapted to such agronomic strategies supporting sustainable N management but also must allow such strategies to work optimally.
3	Irrigation	(drip) fertigation, where mineral fertilizer is supplied via a drip system. This provides more appropriate amounts of nutrients (e.g., N) and water to the active plant root zone than does broadcast fertilization

3	Tillage	No-tillage farming. This is an agricultural technique for growing crops or pasture without disturbing the soil through tillage.

Note: * the values of reduction on cropland Nr loss are the aggregated amount of NH_3, NO_x, N_2O, N leaching, and runoff based on the meta-analysis.

Table S2 Description of the way implementation cost and benefits of the different mitigation measures are calculated and their barriers for application.

Tier	Measures	Implementation cost	Benefits	Viable regions	Inclusion/Barriers	Reference
1	EEF	Prices for slow/controlled-release fertilizers range from a 50% to a 1200% premium over the price of a traditional N fertilizer, Prices for nitrification and urease inhibitors are 8 to 100% premium above the price of traditional N fertilizer	The net benefit to farmers using this practice is estimated to range from $9–$87 per acre estimated from the higher yield.	Areas that rely heavily on urea fertilization, like China, India	farmers' poor access to information, lack of business concept in agriculture, and cost involved in the technology (for smallholder farmers)	[24-27]
1	Amendment	The costs associated with soil amendments can be determined by several factors. This includes • Type and quality of the material • Water content of the material • Freight costs based on distance • Spreading/incorporation costs, which depend on • application rates • type of compost • machinery required – traveling time • scale of the work. It is important to also consider labor and financial costs associated with other practices	Organic amendments increase soil organic matter content and offer many benefits. Over time, organic matter improves soil aeration, water infiltration, and both water- and nutrient-holding capacity. Many organic amendments contain plant nutrients and act as organic fertilizers, therefore	countries in Africa (sub-Saharan Africa), Asia, and Latin America	commercially available amendments may higher in price. public acceptance, Not consistently regulated; Variable quality; Not routinely treated for pathogen reduction; High nutrient loadings in some settings;	[28-30]

		that may need to change in conjunction with amendment application, such as tillage, nutrition, irrigation and crop protection requirements.	could reduce chemical fertilizer input.			
1	Legume	Legume rotations require less N input while providing higher yields, and gross margins, therefore assuming negative implementation cost.	reduce the demand for labor for weed control in subsequent cereal crops (Vereijken and Kloen 1994). In addition, legumes reduce soil erosion (Lawson et al. 2007) and enhance stability and resilience.	can remain economically viable options for farmers, particularly in the dry areas where soil N is limiting, such as Sub-Saharan Africa	farmers' poor access to information, lack of business concept in agriculture	[29, 31-33]
1	Buffer Zone	219-1578 €/kg or 291 €/ha/yr	could also reduce GHG emissions and create a favorable environment for the return of the C sink function, which is characteristic of well-functioning organic soils; avoiding the cost of deposited sediment removal yields annual benefits at the sub-catchments scale.	Buffer zones are important components of a farm’s organic system plan. Buffer zones are put in place to make sure that prohibited substances do not contaminate organic crops.	Land Use Conflict, farmers' poor access to information, lack of business concepts in agriculture,	[34-37]

2	Rate	Advanced soil testing with variable rate technology (VRT) map building was assumed at $8/acre based on industry information.	Increase NUE.	Regions with excessive use of N fertilizer like China India, the EU and the USA.	Soil test, knowledge, negative economic impacts of N management policies on the fertilizer industry.	24, 38-40
2	Type	Urea's average price at $596 / t. Ammonium Sulphate's recent price at $425/t; Ammonium Phosphate's average price at $723/t	Yield increases by 5–11%.	Areas that rely heavily on urea fertilization, like China, India	farmers may have to learn new practices or purchase new technologies to maintain their yield level when significant reduction in N application rate and a change in fertilizer type	
2	Time	Additional applications, especially late-season work, take additional equipment, manpower, and time.	By postponing a portion of the N treatment until the crop is better able to utilize the nutrient, plants take up the N more quickly and efficiently. That means growers get more from their fertilizer investment and fertilizer losses that can contribute	applicable to both smallholder farmers and larger-scale operations.	roadblocks that stand in the way of wider adoption of split programs: (1) Resources. Additional applications, especially late-season work, take additional equipment, manpower and time. (2)	

			to environmental concerns are lessened.		Convenience. (3) weather conditions	
2	Placement	Mechanized deep placement of fertilizer could save some expensive labor because of high efficiency.	deep placement significantly decreased floodwater NH_4^+-N concentration and NH_3 volatilization compared to surface application.	The placement technology is best suited to conditions where the predominant N loss mechanism is ammonia volatilization rather than leaching or denitrification, for example, the rainfed lowland areas,	Small farm size; strongly influenced by weather conditions, and have a high degree of uncertainty	
3	New Cultivar	using improved crop varieties is one of the most effective economical means to improve NUE and stabilize yield. Potential cost includes the infrastructure investment	help boost the productivity of staple crops through genetic improvement.	developing countries where a large yield gap exists, such as Sub-Saharan Africa	The major bottleneck of New Cultivar is the time it takes to develop an improved crop variety. Traditionally, it can take one or two	41-44

					decades because of the many steps of crossing, selection, and testing required. Besides, ongoing investment from the public and private sectors is necessary to maintain the development of high NUE cultivar	
3	Irrigation	A drip irrigation system costs $2,150 per acre on average, with a typical range of $1,800 to $2,500. Costs associated with installing and maintaining drip irrigation.	water conservation as well as saving time, money and hassles.	Drip irrigation can be applied to the irrigation of fruit trees, vegetables, cash crops, and greenhouses. It can also be used for the irrigation of field crops in places with drought and water shortages	the lower profit from crops cultivated using fertigation systems is the main factor restricting their use. Financial aid is a win-win solution to solve the problems of high production costs. Policies and financial programs are required to support the installation of fertigation technology.	45, 46

3	Tillage	No-till farming requires some different skills than conventional farming. A combination of technique, equipment, pesticides, crop rotation, fertilization, and irrigation have to be used for local conditions. Generally, it reduces annual 2/3 fuel and labor investments compared to conventional tillage.	Saving time and improving soil health, lead to additional economic benefits.	applicable mainly in sandy and dry soils on sloping terrain	To ensure the successful transition from conventional to no-tillage or minimum tillage systems, nutrient management considerations including starter fertilizer and timing of nutrients application are critical. A proper fertilizer program is necessary	[47-49]

Table S3 Data sources used for assessing global and regional cropland N budgets and mitigation efficiency with assumptions about data variation and type of uncertainty

Item	Unit	CV	Uncertainty type	Importance	Data source
**Activity data**					
Cropland fertilizer use	Gg N yr^{-1}	10%	U	*****	FAO[10], IFA[7]
Cultivated areas	ha	10%	U	*****	FAO[6]
Crop yield	t/ha	10%	U	*****	FAO[6]
Irrigation water volume	1000 ML yr^{-1}	20%	U	****	FAO[6]
Irrigated area	ha	10%	U	***	ICID[50]
Manure production	Gg N yr^{-1}	10	U	****	
**Parameters**					
Biological N fixation rate	kg N ha^{-1} yr^{-1}	25%	N	****	Zhang et al. (2015)[51]
Crop N content (grain)	%	20%	N	****	CHANS, IMAGE, MAgPIE
Crop N content (straw)	%		N	****	
Harvest index	-		N	***	
Crop residue fate	%	25%	U	***	FAO[6]
N concentration of irrigation	mg N L^{-1}	25%	U	***	FAO[6]
N deposition rate	mg N m^{-2} yr^{-1}	50%	U	****	FAO[6]
Manure recycling ratio	%	25%	U	****	FAO[6]
**Emission factors**					
N_r to air (NH_3, N_2O, NO_x, N_2)	% or kg N ha^{-1}	50%	N	*****	CHANS, IMAGE, MAgPIE
N_r to water (NO_3^{-1} & other N_r)	% or kg N ha^{-1}	50%	N	*****	CHANS, IMAGE, MAgPIE
**Mitigation options**					
Mitigation efficiency	%	See Figure F4-F14	N	*****	Meta-analysis. References listed in Supplementary Data S10

Adoption rate	%	30%	U	*****

Note: CV means the coefficients of variation for the main input data. U and N represent the Uniform distribution and Normal distribution, respectively. The numbers of stars in the ‘Importance’ column indicate the importance of the parameter in determining the N budget, which also determines the priority for further development.

Table S4 Uncertainty analysis

	Unit	Calculated value	Estimated range	Normalized range (%)	Standard deviation	Uncertainty contribution (%)
Global cropland N budget in current 2015						
N input	(Tg N yr^{-1})	200	157-233	19%	37.5	-
Fertilizer N use	(Tg N yr^{-1})	105	92-116	12%	12	32%
Manure N	(Tg N yr^{-1})	27	21-33	22%	6	16%
Deposition N	(Tg N yr^{-1})	16	10-19	31%	4.5	12%
BNF N	(Tg N yr^{-1})	44	30-57	31%	13.5	36%
Irrigation N	(Tg N yr^{-1})	9	8-11	16%	1.5	4%
N harvest	(Tg N yr^{-1})	85	76-93	10%	8.5	-
NUE	%	42%	39-45%	14%	6%	-
N surplus	(Tg N yr^{-1})	116	85-143	37%	42	-
NH_3 emission	(Tg N yr^{-1})	20	15-27	29%	6	14%
N_2O emission	(Tg N yr^{-1})	4	2-5	37%	1	3%
NO_x emission	(Tg N yr^{-1})	5	3-6	33%	2	4%
N leaching and runoff	(Tg N yr^{-1})	52	30-72	41%	21	50%
N_2 emission	(Tg N yr^{-1})	35	23-47	34%	12	29%
Global cropland N budget under optimized 2015						
N input	(Tg N yr^{-1})	180	148-219	19%	36	-

Fertilizer N use	(Tg N yr^{-1})	83	70-94	15%	12	34%
Manure N	(Tg N yr^{-1})	37	32-42	14%	5	14%
Deposition N	(Tg N yr^{-1})	10	7-14	33%	4	10%
BNF N	(Tg N yr^{-1})	44	31-57	30%	13	37%
Irrigation N	(Tg N yr^{-1})	9	8-12	20%	2	6%
N harvest	(Tg N yr^{-1})	102	91-113	11%	11	-
NUE	%	55%	48-61%	12%	0	-
N surplus	(Tg N yr^{-1})	82	57-106	30%	25	-
NH_3 emission	(Tg N yr^{-1})	13	8-17	36%	5	18%
N_2O emission	(Tg N yr^{-1})	2	1-3	50%	1	4%
NO_x emission	(Tg N yr^{-1})	3	2-5	43%	2	6%
N leaching and runoff	(Tg N yr^{-1})	36	22-50	39%	14	57%
N_2 emission	(Tg N yr^{-1})	27	20-34	26%	7	29%
Global costs and benefits under optimized 2015						
Net Implementation cost	(Billion USD)	19	15-25	25%	5	-
fertilizer saving cost	(Billion USD)	15	12-19	23%	4	-
Total benefit	(Billion USD)	533	400-650	24%	125	-
Ecosystem benefits	(Billion USD)	152	112-183	24%	36	28%
Health benefits	(Billion USD)	130	93-175	31%	41	33%

Yield benefits	(Billion USD)	253	198-293	19%	47.5	38%
Climate impact	(Billion USD)	-2	-3--1	-50%	1	1%
Net social benefit	(Billion USD)	514	385-625	24%	120	-

Note: current 2015 refers to the current status of the global cropland N budget, while optimized 2015 represents the new cropland N budget with the optimized adoption of the selected package of measures. The uncertainty range stands for the 95% confidence interval. The uncertainty contribution ranges from 0 (no contribution) to 100% (very large contribution). “-” means not applicable or not considered relevant.

Table S5 Determination criteria of the potential implementation rate for different tiered mitigation measures in the tiered scenario setting

Options	Determination criteria	Range				
**EEF**	IF Fertilizer N application rate (kg N/ha/yr)	<30	30<x<100	100<x<200	200<x<400	>400
	EEF adoption rate (%)	10-20%	20-40%	40-60%	60-80%	80-100%
**Organic amendment**	IF Manure N application rate (kg N/ha/yr)	<20	20<x<40	40<x<60	60<x<100	>100
	Organic amendment adoption rate (%)	50-60%	40-50%	30-40%	20-30%	0-20%
**Legume rotation**	IF BNF rate (kg N/ha/yr)	<10	10<x<20	20<x<30	30<x<40	>40
	Legume rotation adoption rate (%)	50%	40-50%	30-40%	20-30%	0-20%
**Buffer zone implementation rate (%)**	IF cropland N input density (kg N/ha/yr)	<50	50<x<100	100 <x< 200	200 <x< 400	>400
	Low-income nation	5-10%	10-20%	20-30%	30-35%	35%
	Lower-middle-income nation	20-30%	30-40%	40-45%	45-50%	50%
	Upper-middle-income nation	30-50%	50-60%	60-70%	70-80%	80%
	High-income nation	40-60%	60-80%	80-90%	90-100%	100%
**4R implementation rate (%)**	IF cropland N input density (kg N/ha/yr)	<100	100<x<200	200 <x< 300	300 <x< 500	>500
	Low-income nation	10-20%	20-30%	30-40%	40-50%	50-60%
	Lower-middle-income nation	30-40%	40-50%	50-55%	55-60%	>60%

	Upper-middle-income nation	40-50%	50-60%	60-70%	70-80%	80%
	High-income nation	40-60%	60-80%	80-90%	90-100%	100%
**New cultivars**	IF cropland N harvest rate (kg N/ha/yr)	<40	40<x<60	60<x<80	80<x<100	>100
	New cultivar promotion rate (%)	50-60%	40-50%	30-40%	20-30%	0-20%
**Improved irrigation**	IF cropland N harvest rate (kg N/ha/yr)	<40	40<x<60	60<x<80	80<x<100	>100
	(% adoption)	40-50%	30-40%	20-30%	10-20%	0-10%
**No tillage**	IF cropland N leaching and runoff rate (kg N/ha/yr)	<10	10<x <50	50<x<80	80<x<120	>120
	No tillage adoption rate (%)	0-10%	10-20%	20-30%	30-40%	40-50%

Note: the adoption rate of a specific option for a certain nation/region is determined by the current different N input density and its source contribution (fertilizer N rate, manure N rate, BNF rate). N loss intensity and the social-economic level in a specific nation/region will also affect the adoption rate of mitigation options.

Table S6 Correlation coefficient of impacts of mitigation measures on different N use/loss in different countries

Country \ Benchmark	Australia	Bangladesh	Brazil	Canada	China	France	Germany	India	Indonesia	Iran
**Australia**		-11.20	12.12	-4.76	-6.83	6.12	11.11	-14.27	-12.55	8.72
**Bangladesh**	11.20		23.32	6.44	4.36	17.32	22.31	-3.07	-1.36	19.92
**Brazil**	-12.12	-23.32		-16.88	-18.95	-6.00	-1.01	-26.39	-24.67	-3.40
**Canada**	4.76	-6.44	16.88		-2.08	10.88	15.87	-9.51	-7.80	13.47
**China**	6.83	-4.36	18.95	2.08		12.95	17.94*	-7.44	-5.72	15.55**
**France**	-6.12	-17.32	6.00	-10.88	-12.95		4.99	-20.39	-18.67	2.60
**Germany**	-11.11	-22.31	1.01	-15.87	-17.94*	-4.99		-25.38**	-23.66	-2.39
**India**	14.27	3.07	26.39	9.51	7.44	20.39	25.38**		1.72	22.99**
**Indonesia**	12.55	1.36	24.67	7.80	5.72	18.67	23.66	-1.72		21.27
**Iran**	-8.72	-19.92	3.40	-13.47	-15.55**	-2.60	2.39	-22.99	-21.27	
**Italy**	9.94	-1.26	22.06	5.18	3.11	16.06	21.05*	-4.33	-2.61	18.66*
**Kenya**	48.62*	37.42	60.74	43.86	41.79	54.74	59.73*	34.35	36.07	57.34*
**Mexico**	-46.63	-57.83*	-34.51	-51.39*	-53.46*	-40.51	-35.52	-60.90**	-59.18	-37.91
**Philippines**	9.77	-1.43	21.89	5.01	2.94	15.89	20.88	-4.50	-2.78	18.49
**Spain**	8.11	-3.08	20.23	3.36	1.28	14.23	19.22*	-6.16	-4.44	16.83*
**Sweden**	14.60	3.40	26.72	9.84	7.77	20.72	25.71*	0.33	2.05	23.32**
**Thailand**	-3.20	-14.40	8.92	-7.96	-10.04	2.91	7.90	-17.48	-15.76	5.51
**UK**	5.05	-6.14	17.17	0.30	-1.78	11.17	16.16	-9.22	-7.50	13.77
**USA**	7.32	-3.87	19.44	2.57	0.49	13.44	18.43*	-6.95	-5.23	16.04*
**Zimbabwe**	11.12	-0.08	23.24	6.36	4.29	17.24	22.23	-3.15	-1.43	19.84

Benchmark / Country	Italy	Kenya	Mexico	Philippines	Spain	Sweden	Thailand	UK	USA	Zimbabwe
**Australia**	-9.94	-48.62*	46.63	-9.77	-8.11	-14.60	3.20	-5.05	-7.32	-11.12
**Bangladesh**	1.26	-37.42	57.83*	1.43	3.08	-3.40	14.40	6.14	3.87	0.08
**Brazil**	-22.06	-60.74	34.51	-21.89	-20.23	-26.72	-8.92	-17.17	-19.44	-23.24
**Canada**	-5.18	-43.86	51.39*	-5.01	-3.36	-9.84	7.96	-0.30	-2.57	-6.36
**China**	-3.11	-41.79	53.46*	-2.94	-1.28	-7.77	10.04	1.78	-0.49	-4.29
**France**	-16.06	-54.74	40.51	-15.89	-14.23	-20.72	-2.91	-11.17	-13.44	-17.24
**Germany**	-21.05*	-59.73*	35.52	-20.88	-19.22*	-25.71*	-7.90	-16.16	-18.43*	-22.23
**India**	4.33	-34.35	60.90**	4.50	6.16	-0.33	17.48	9.22	6.95	3.15
**Indonesia**	2.61	-36.07	59.18	2.78	4.44	-2.05	15.76	7.50	5.23	1.43
**Iran**	-18.66*	-57.34*	37.91	-18.49	-16.83*	-23.32**	-5.51	-13.77	-16.04*	-19.84
**Italy**		-38.68	56.57*	0.17	1.82	-4.66	13.14	4.89	2.61	-1.18
**Kenya**	38.68		95.25***	38.85	40.50	34.02	51.82*	43.57	41.29	37.50
**Mexico**	-56.57*	-95.25***		-56.40	-54.74*	-61.23**	-43.43	-51.68	-53.95*	-57.75*
**Philippines**	-0.17	-38.85	56.40		1.65	-4.83	12.97	4.72	2.44	-1.35
**Spain**	-1.82	-40.50	54.74*	-1.65		-6.49	11.32	3.06	0.79	-3.01
**Sweden**	4.66	-34.02	61.23**	4.83	6.49		17.80	9.55	7.28	3.48
**Thailand**	-13.14	-51.82*	43.43	-12.97	-11.32	-17.80		-8.26	-10.53	-14.32
**UK**	-4.89	-43.57	51.68	-4.72	-3.06	-9.55	8.26			-6.07
**USA**	-2.61	-41.29	53.95*	-2.44	-0.79	-7.28	10.53	2.27	-2.27	-3.80
**Zimbabwe**	1.18	-37.50	57.75*	1.35	3.01	-3.48	14.32	6.07	3.80	

This table lists the coefficient of each country to the dependent variable Y (*response*) after controlling the independent variables of strategy type and N paths during regression analysis. Each column reflects the country's relative coefficient to Y when compared to the benchmark country. The goal of establishing different benchmarks is to compare the difference of every two countries responding to the dependent variable. Stars indicate

statistical significance of coefficient: $^{*}p \leq 0.05$, $^{**}p \leq 0.01$, $^{***}p \leq 0.005$. If the coefficient has no star, it indicates that there is no significant difference between the country and the benchmark country of responses of strategies. The results of this table show that under the same strategy type and N path, only a small proportion of countries (such as Mexico) have significantly different effects of strategies than other countries. In other words, there is no significantly different effect of strategies across most countries.

Supplementary Figures

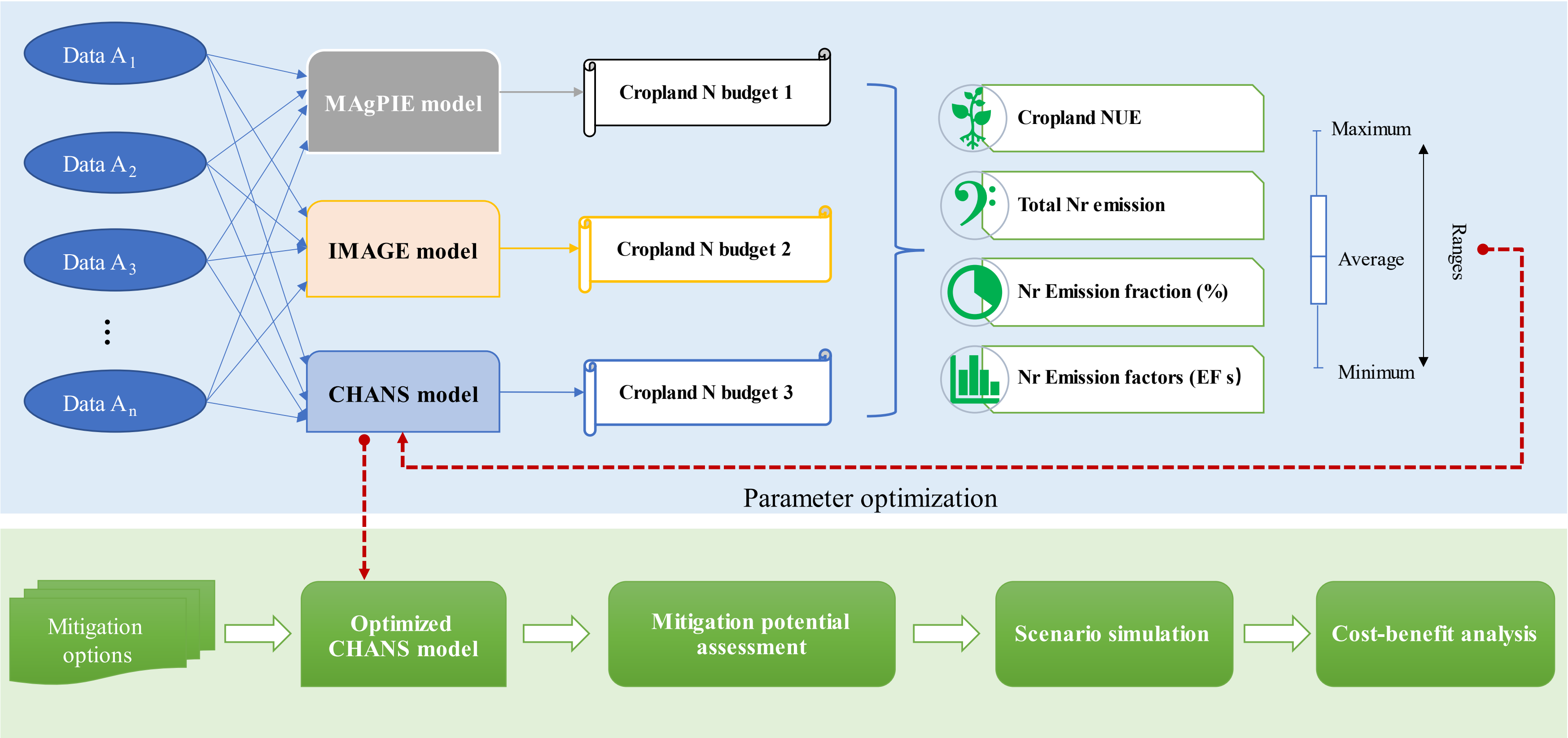

Figure S1 | Compilation of global cropland N budgets based on multi-model combination

Here we compared the N_r related parameters extracted from the three models and get the average-weighted value back only to the CHANS model to improve the robustness and reliability of the CHANS model and then perform the mitigation assessment of the potential, cost, and benefits under different scenarios.

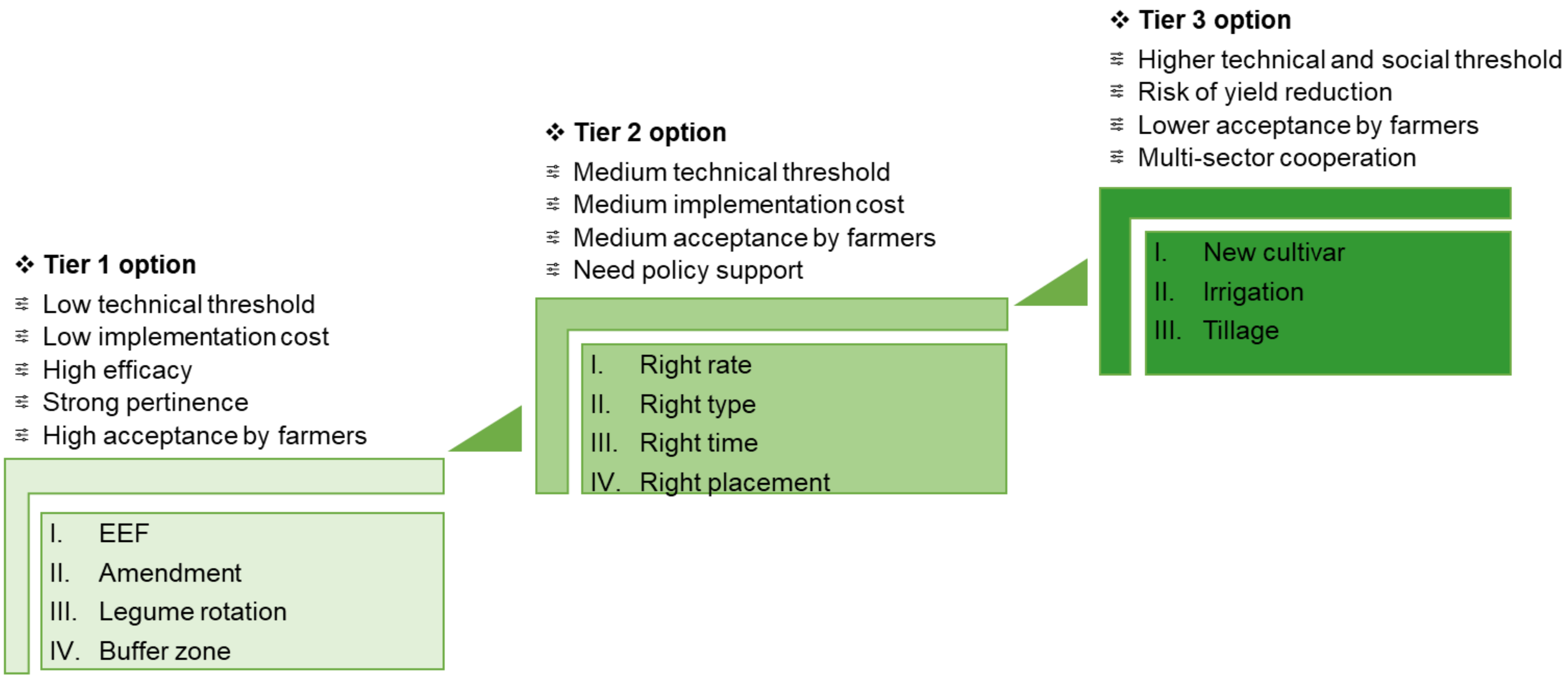

Figure S2 | Classification criteria for three tiers of mitigation measures adopted in this study

Tier 1 mitigation approaches are simple and socially acceptable, while Tier 2 and Tier 3 measures are increasingly more complex, costly, and associated with lower acceptability.

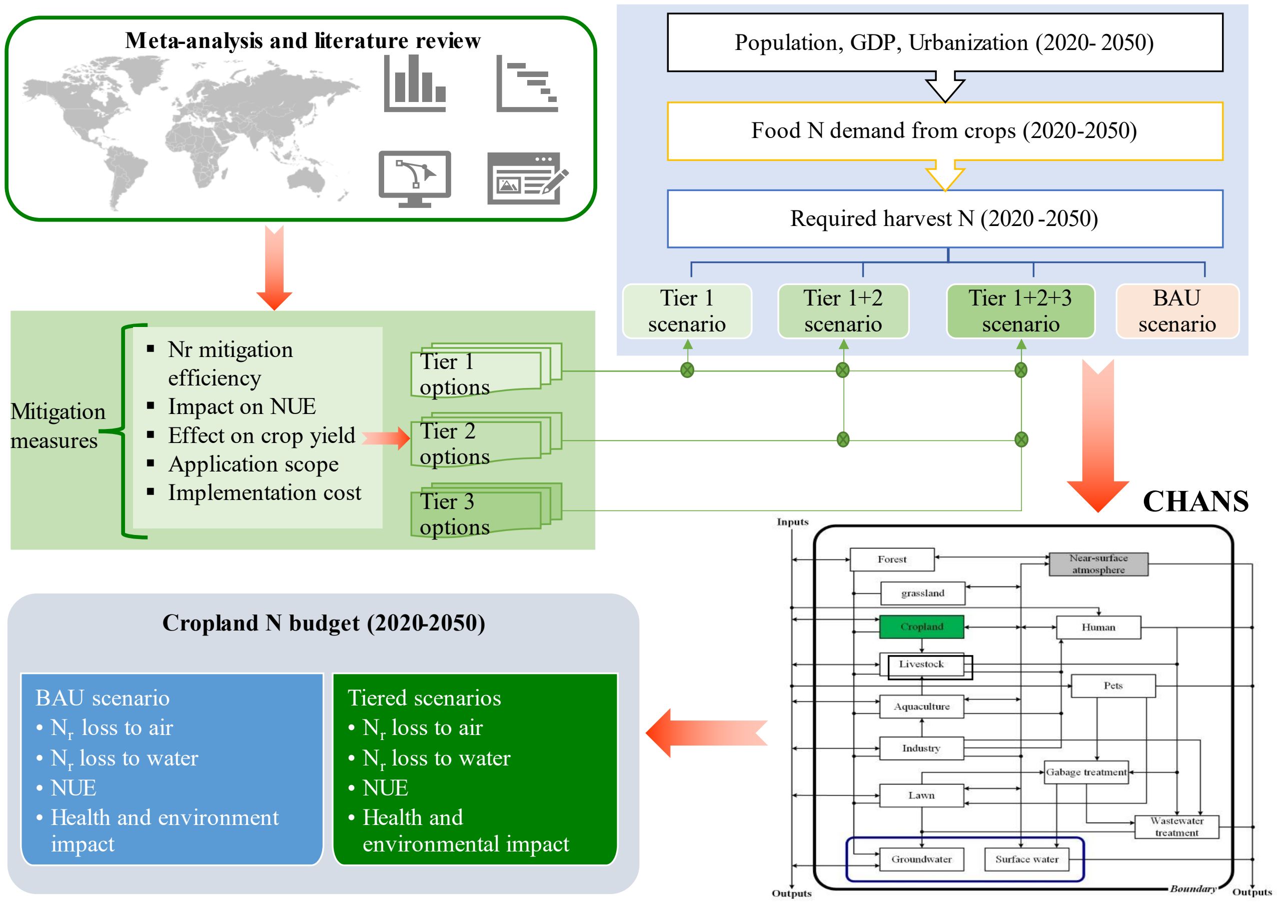

Figure S3 | Integration of meta-analysis with CHANS model to perform scenario analysis

BAU stands for the business-as-usual scenario without any mitigation measures, Tiered scenarios include Tier 1, Tier 1+2, and Tier 1+2+3 Scenario, which are endowed with related tiered options to compare their mitigation potential and environmental outcomes.

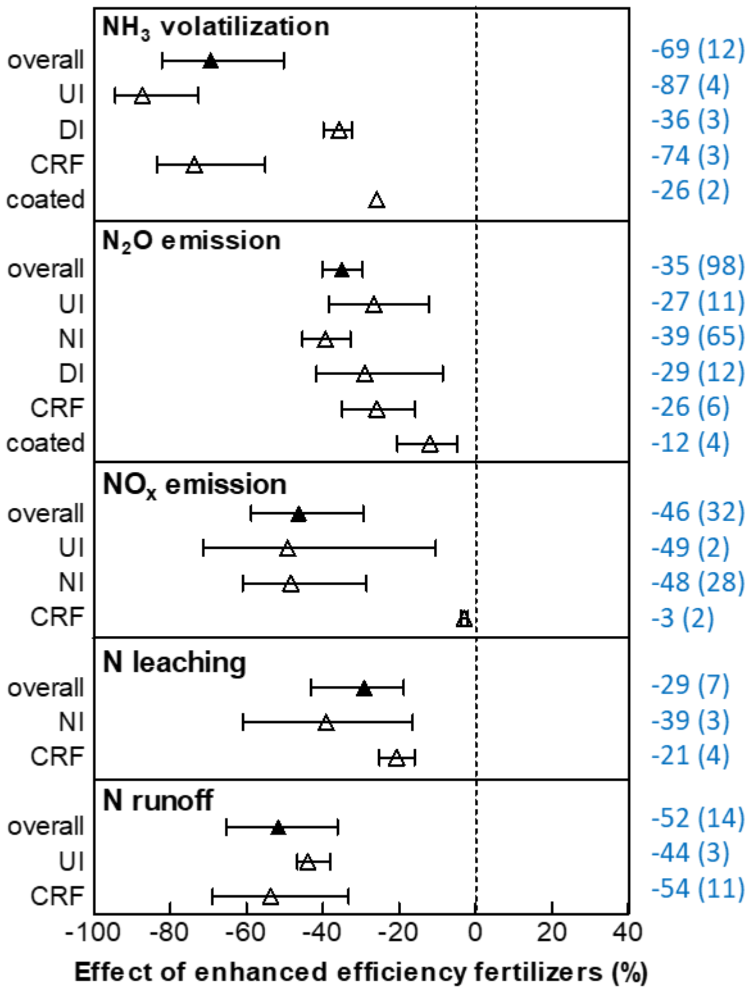

Figure S4 | Effects of enhanced efficiency fertilizers on N_r loss from croplands.
The figure shows the effects of enhanced efficiency fertilizers on the amount of N loss via different pathways (presented as a percentage change). The results are presented as the mean and 95% confidence intervals of all pooled data of using enhanced efficiency fertilizers (shown as ‘overall’), and according to the type of these fertilizers (UI: urease inhibitor; NI: nitrification inhibitor; DI: dual inhibitor (a combination of UI and NI); CRF: controlled-release fertilizer; or coated: coated fertilizer), as compared with a fertilizer (mostly granular urea) without the inhibitor or coating. Effects were significant ($p < 0.05$) if the confidence intervals did not overlap with zero. Negative values indicate a reduction in the amount of N losses due to the treatment whereas positive values indicate an increase in losses. Blue numbers show the exact mean value of change, with the sample size in brackets.

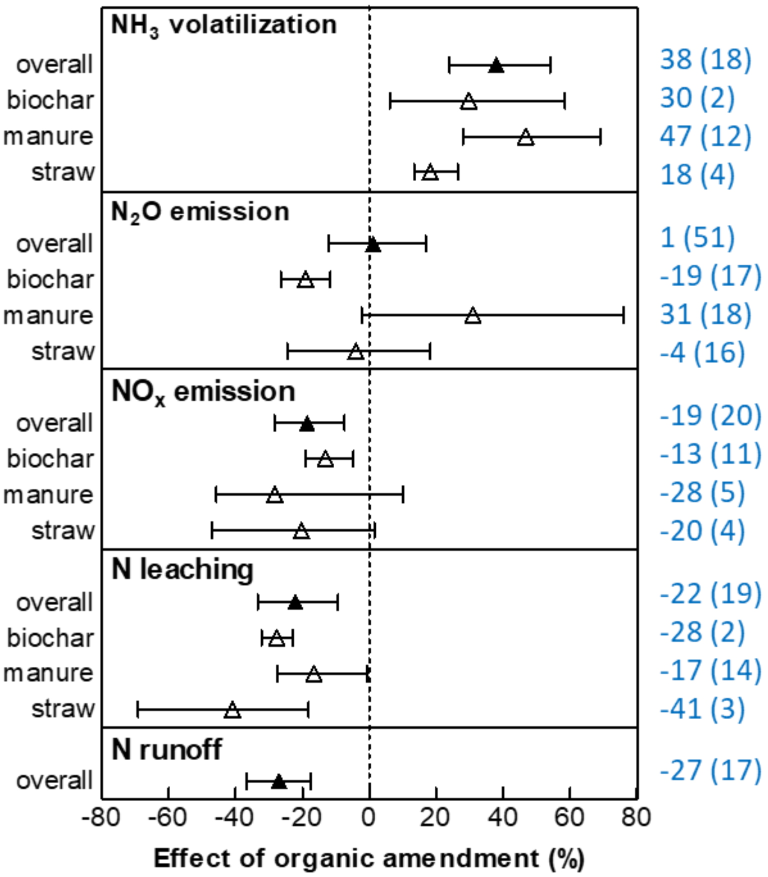

Figure S5 | Effects of organic amendment on N_r loss from croplands.

The figure shows the effects of organic amendment on the amount of N loss via different pathways (presented as a percentage change). The results are presented as the mean and 95% confidence intervals of all pooled data (shown as 'overall'), and according to organic amendment types (biochar, manure or straw), as compared with a reference without amendment. Effects were significant (p < 0.05) if the confidence intervals did not overlap with zero. Negative values indicate a reduction in the amount of N losses due to the treatment whereas positive values indicate an increase in losses. Blue numbers show the exact mean value of change, with the sample size in brackets.

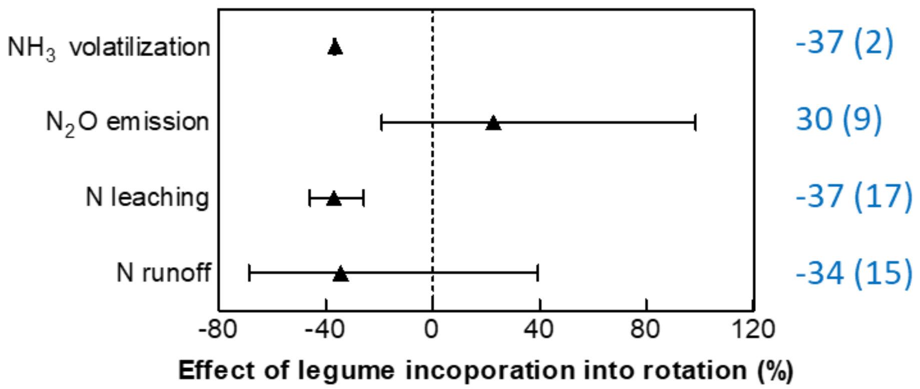

Figure S6 | Effects of legume rotation on N_r loss from croplands.
The figure shows the effects of the incorporation of a legume in a rotation on the amount of N loss via different pathways (presented as a percentage change). The results are presented as the mean and 95% confidence intervals of all pooled data of incorporating a legume into a rotation, as compared with a rotation without a legume. Only crop rotations of at least two years are included, where one year of the rotation was planted with a legume crop, relative to a non-legume crop; other crops grown in the rotation were the same between the treatment and the control. Effects were significant ($p < 0.05$) if the confidence intervals did not overlap with zero. Negative values indicate a reduction in the amount of N losses due to the treatment whereas positive values indicate an increase in losses. Blue numbers show the exact mean value of change, with the sample size in brackets.

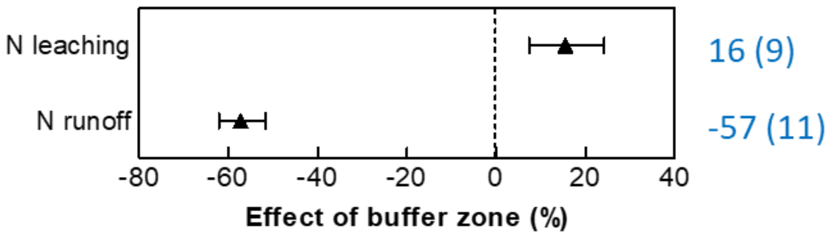

Figure S7 | Effects of the buffer zone on N_r loss from croplands.
The figure shows the effects of buffer zone on the amount of N loss via different pathways (presented as a percentage change). The results are presented as the mean and 95% confidence intervals of all pooled data of the use of buffer zone, as compared with a reference without a buffer zone. From the database, the buffer zone was either 4 m^3 or 150 m^3, with the following types: grass strips (different species), tree strips, grass barrier, Leucaena hedge. Effects were significant ($p < 0.05$) if the confidence intervals did not overlap with zero. Negative values indicate a reduction in the amount of N losses due to the treatment whereas positive values indicate an increase in losses. Blue numbers show the exact mean value of change, with the sample size in brackets.

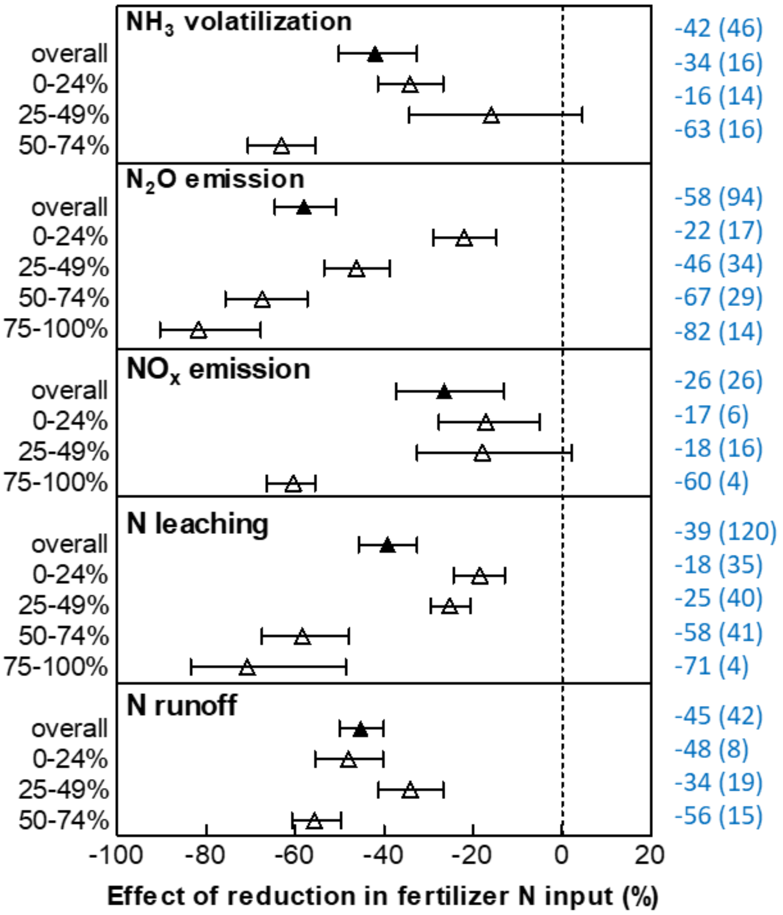

Figure S8 | Effects of right rate of N fertilizer application (reduction) on N_r loss from croplands.

The figure shows the effects of reduction in fertilizer N input on the amount of N loss via different pathways (presented as a percentage change). The results are presented as the mean and 95% confidence intervals of all pooled data (shown as 'overall'), and according to the percentage of N input reduction (0-24, 25-49, 50-74, or 75-100%), as compared with the highest fertilizer input rate used in a study. Effects were significant ($p < 0.05$) if the confidence intervals did not overlap with zero. Negative values indicate a reduction in the amount of N losses due to the treatment whereas positive values indicate an increase in losses. Blue numbers show the exact mean value of change, with the sample size in brackets.

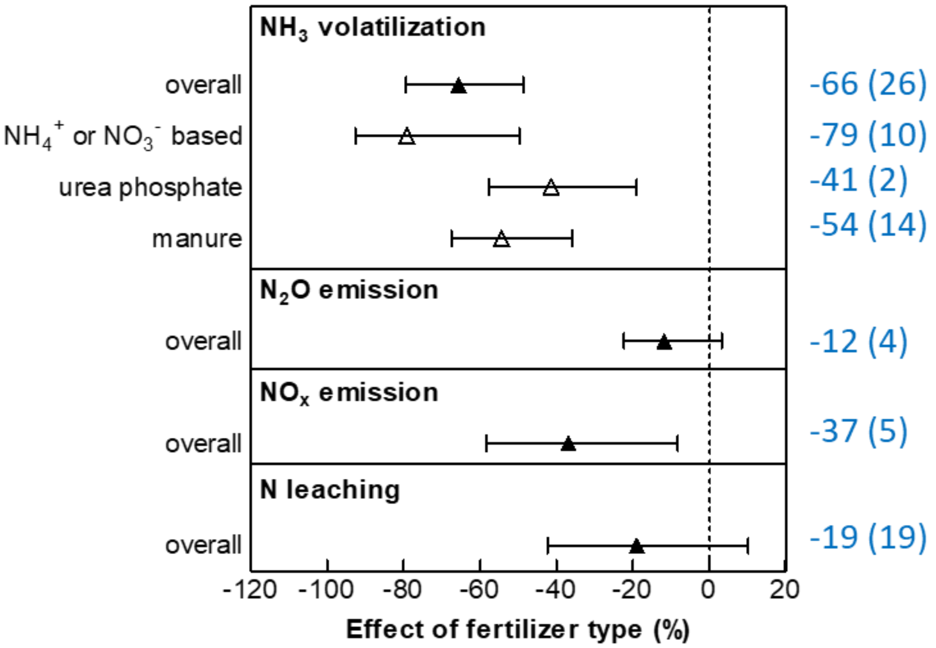

Figure S9 | Effects of the fertilizer type on N_r loss from croplands

The figure shows the effects of fertilizer type on the amount of N loss via different pathways (presented as a percentage change). The results are presented as the mean and 95% confidence intervals of all pooled data (shown as 'overall'), and according to the fertilizer type (NH_4^+ or NO_3^- based, urea phosphate, or manure), as compared with urea as a reference. The method of N application was the same for the treatment and the control. Effects were significant ($p < 0.05$) if the confidence intervals did not overlap with zero. Negative values indicate a reduction in the amount of N losses due to the treatment whereas positive values indicate an increase in losses. Blue numbers show the exact mean value of change, with the sample size in brackets.

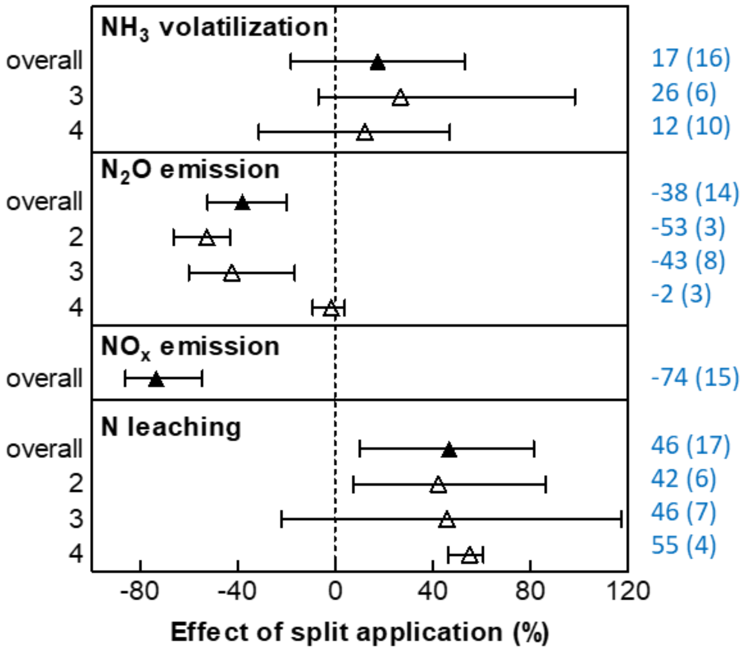

Figure S10 | Effects of the N fertilizer time in terms of split application of N fertilizers on N_r loss from croplands.

The figure shows the effects of split application of fertilizer on the amount of N loss via different pathways (presented as a percentage change). The results are presented as the mean and 95% confidence intervals of all pooled data (shown as ‘overall’), and according to total fertilizer N addition split into 2, 3 or 4 doses, as compared with a reference where all N is added in one dose. Effects were significant ($p < 0.05$) if the confidence intervals did not overlap with zero. Negative values indicate a reduction in the amount of N losses due to the treatment whereas positive values indicate an increase in losses. Blue numbers show the exact mean value of change, with the sample size in brackets.

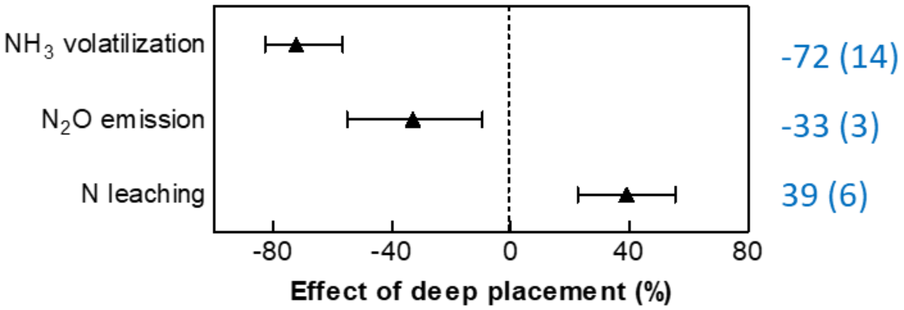

Figure S11 | Effects of N fertilizer place, in terms of deep placement on N_r loss from croplands.

The figure shows the effects of deep placement of fertilizer on the amount of N loss via different pathways (presented as a percentage change). The results are presented as the mean and 95% confidence intervals of all pooled data of deep placement of fertilizer, as compared with topdressing of the same fertilizer type. Effects were significant ($p < 0.05$) if the confidence intervals did not overlap with zero. Negative values indicate a reduction in the amount of N losses due to the treatment whereas positive values indicate an increase in losses. Blue numbers show the exact mean value of change, with the sample size in brackets.

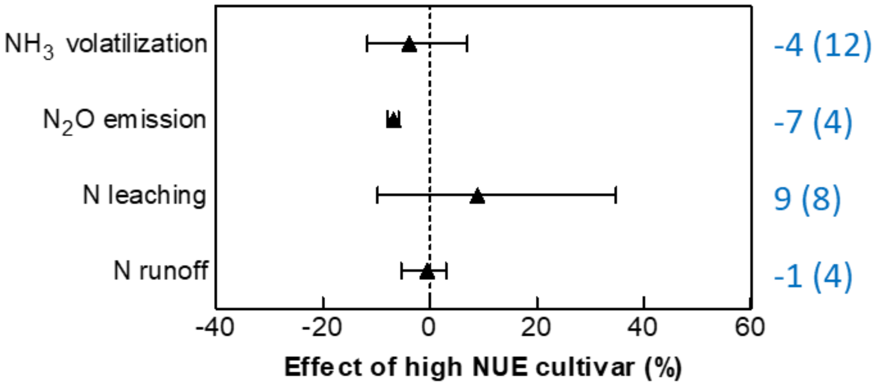

Figure S12 | Effects of high NUE cultivar on Nr loss from croplands.

The figure shows the effects of using high NUE cultivar on the amount of N loss via different pathways (presented as a percentage change). The results are presented as the mean and 95% confidence intervals of all pooled data of using high NUE cultivar, as compared with a low NUE cultivar. Effects were significant ($p < 0.05$) if the confidence intervals did not overlap with zero. Negative values indicate a reduction in the amount of N losses due to the treatment whereas positive values indicate an increase in losses. Blue numbers show the exact mean value of change, with the sample size in brackets.

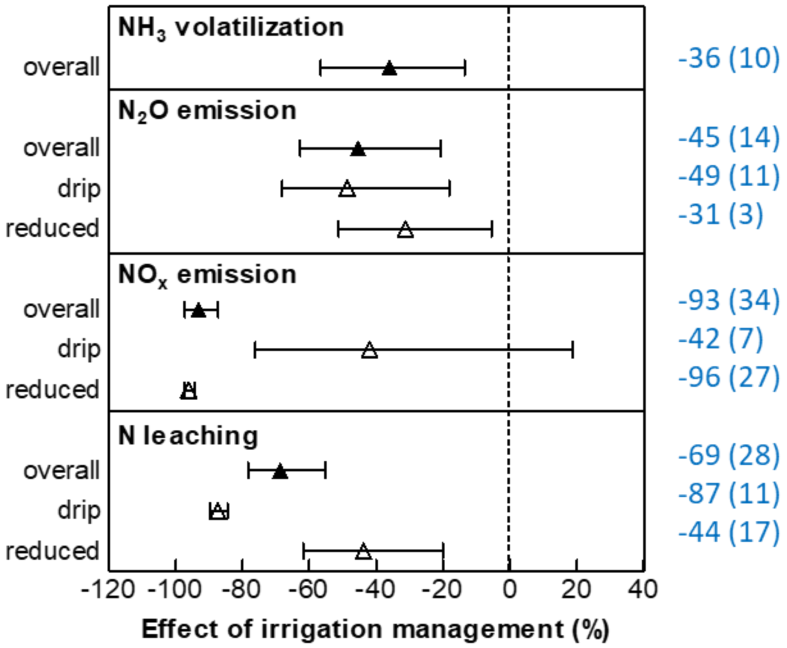

Figure S13 | Effects of reduced irrigation or drip irrigation on N_r loss from croplands. The figure shows the effects of irrigation management on the amount of N loss via different pathways (presented as a percentage change). The results are presented as the mean and 95% confidence intervals of all pooled data (shown as 'overall'), and according to reduced irrigation (shown as 'reduced') or the use of drip irrigation (shown as 'drip'), as compared with the local irrigation practice. Effects were significant ($p < 0.05$) if the confidence intervals did not overlap with zero. Negative values indicate a reduction in the amount of N losses due to the treatment whereas positive values indicate an increase in losses. Blue numbers show the exact mean value of change, with the sample size in brackets.

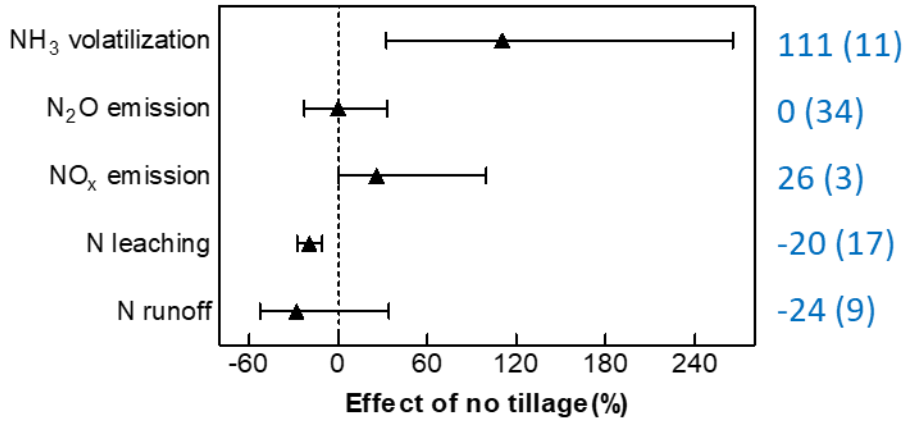

Figure S14 | Effects of no-tillage on N_r loss from croplands.
The figure shows the effects of no-tillage on the amount of N loss via different pathways (presented as a percentage change). The results are presented as the mean and 95% confidence intervals of all pooled data of no-tillage, as compared with conventional tillage. Effects were significant ($p < 0.05$) if the confidence intervals did not overlap with zero. Negative values indicate a reduction in the amount of N losses due to the treatment whereas positive values indicate an increase in losses. Blue numbers show the exact mean value of change, with the sample size in brackets.

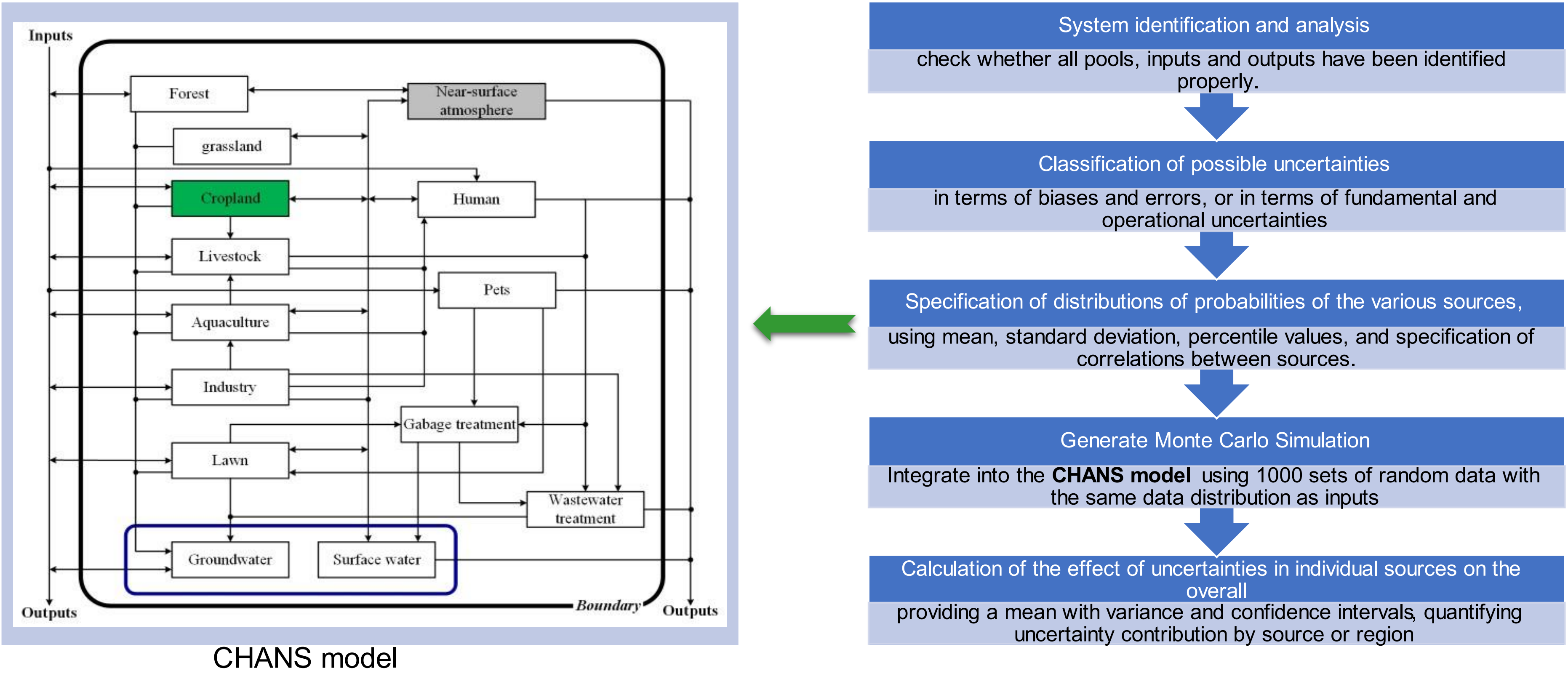

Figure S15 | Steps of uncertainty analysis using Monte Carlo simulation

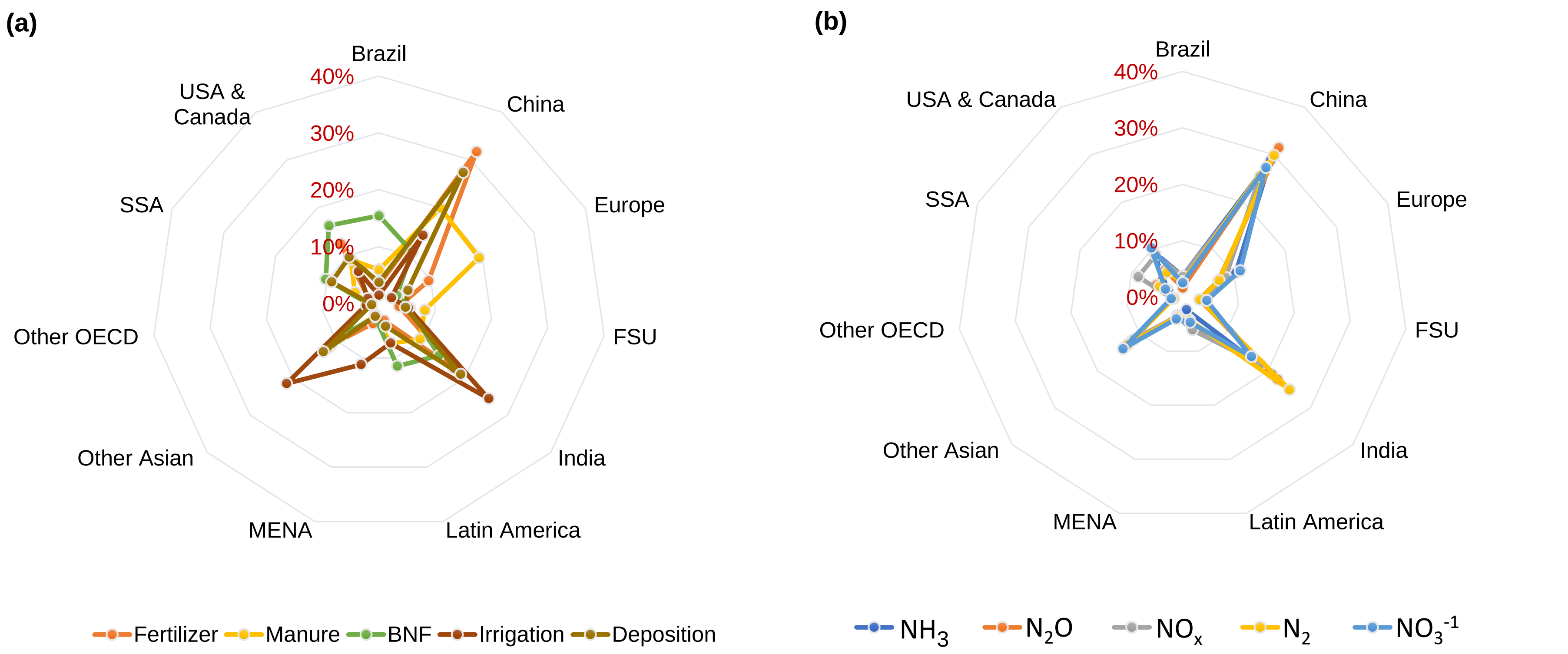

Figure S16 | Uncertainty contribution of cropland N budgets by regions

(a) the regional uncertainty contribution (%) to the global cropland inputs of fertilizer, manure, BNF, irrigation and deposition. (b) the regional uncertainty contribution (%) to the global cropland N surplus, including NH_3, N_2O, NO_x, N_2, NO_3-1(leaching and runoff). FSU, Former Soviet Union; MENA, Middle-East and North Africa; OECD, the Organization for Economic Co-operation and Development, SSA, Sub-Saharan Africa.

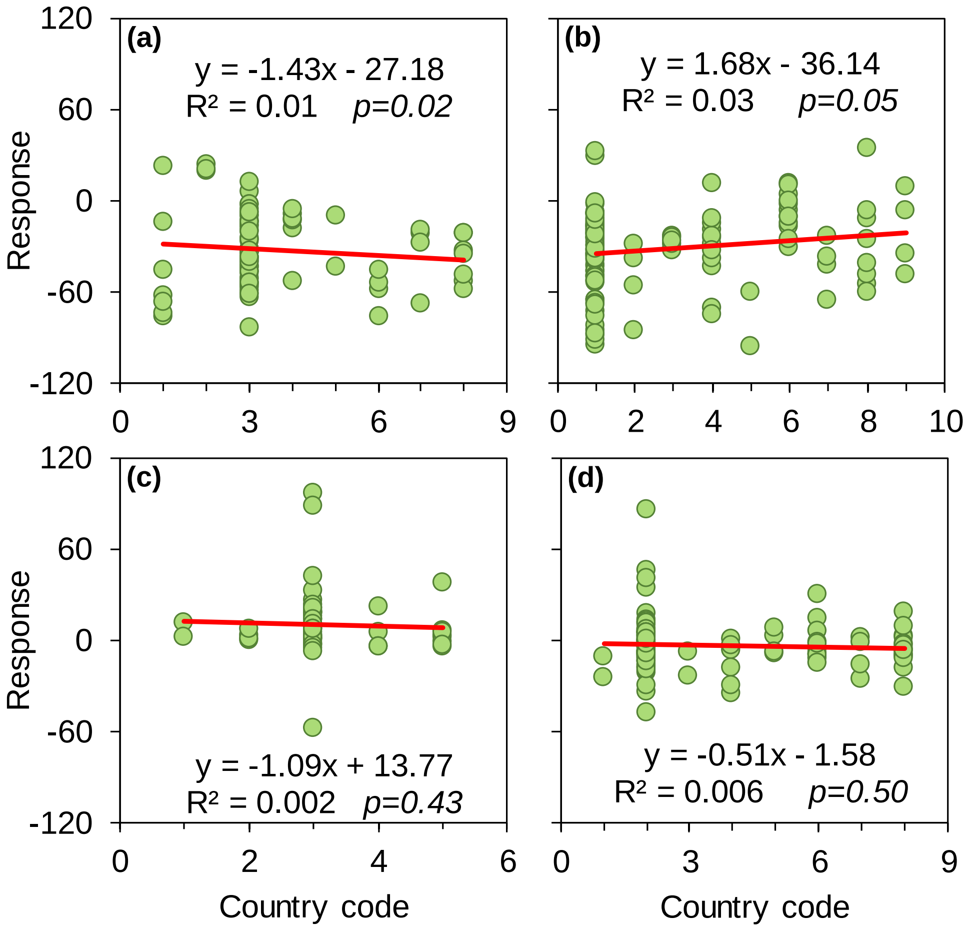

Figure S17 | Impacts N mitigation measures on N use/loss across different countries

(a) EFF by N_2O; (b) Fertilizer rate by leaching; (c) EEF by yield; (d) Fertilizer rate by yield. EEF refers to enhanced efficiency fertilizers. The mitigation response is not significant across countries suggested that the N mitigation measures generally have a consistent effect in different global regions. Only some typical examples are shown here. The overall analysis can be found in Table S6.

References:

1. Sutton, M.A., Howard, C.M., Mason, K.E., Brownlie, W.J. & Cordovil, C.M.D.S. Nitrogen opportunities for agriculture, food & environment. UNECE Air Convention: Guidance Document on Integrated Sustainable Nitrogen Management. (2022).
2. Gu, B., Ju, X., Chang, J., Ge, Y. & Vitousek, P.M. Integrated reactive nitrogen budgets and future trends in China. *Proc. Natl Acad. Sci. USA* **112**, 8792-8797 (2015).
3. Gu, B. & Zhang, X. The Coupled Human And Natural Systems (CHANS) Nitrogen Cycling Model. (2020).
4. EDGAR. Emissions Database for Global Atmospheric Research (EDGAR v5.0). (2020).
5. World Bank. *State and Trends of Carbon Pricing 2020* (Washington, DC: World Bank, 2020).
6. FAOSTAT. Food and Agriculture Organization of the United Nations Online Statistical Databases. (2018).
7. IFADATA. International Fertilizer Industry Association (2018). (2018).
8. Lassaletta, L., Billen, G., Grizzetti, B., Anglade, J. & Garnier, J. 50 year trends in nitrogen use efficiency of world cropping systems: the relationship between yield and nitrogen input to cropland. *Environ Res Lett* **9**, 105011 (2014).
9. Schulte-Uebbing, L. & de Vries, W. Reconciling food production and environmental boundaries for nitrogen in the European Union. *Sci Total Environ* **786**, 147427 (2021).
10. FAOSTAT. Soil nutrient budget: Global, regional and country trends, 1961–2018. (FAOSTAT, 2020).
11. Bouwman, A.F., *et al*. Lessons from temporal and spatial patterns in global use of N and P fertilizer on cropland. *Sci Rep-Uk* **7** (2017).
12. IPCC. IPCC emission factor database. *Environmental Protection* (2007).
13. PBL. Integrated Model to Assess the Global Environment (IMAGE) model Documentation. (2020).
14. MAgPIE. Model of Agricultural Production and its Impact on the Environment. (2021).
15. Bodirsky, B.L., *et al*. mrcommons: MadRat commons Input Data Library. (Zenodo, 2020).
16. Bodirsky, B.L., *et al*. N_2O emissions from the global agricultural nitrogen cycle: current state and future scenarios. *Biogeosciences* **9**, 4169-4197 (2012).
17. Zhang, X., *et al*. Societal benefits of halving agricultural ammonia emissions in China far exceed the abatement costs. *Nat Commun* **11** (2020).
18. Kriegler, E., *et al*. Fossil-fueled development (SSP5): An energy and resource intensive scenario for the 21st century. *Global Environmental Change* **42**, 297-315 (2017).
19. Riahi, K., *et al*. The Shared Socioeconomic Pathways and their energy, land use, and greenhouse gas emissions implications: An overview. *Global Environmental Change* **42**, 153-168 (2017).
20. Okello, G.O., Ngode, L. & Saina, E. Social factors affecting adoption of zero-grazing dairy farming technology among smallholder farmers in Bondo subcounty, Kenya. *International*

Journal of Agricultural Extension and Rural Development Studies (2019).

21. Dessart, F.J., Barreiro-Hurlé, J. & van Bavel, R. Behavioural factors affecting the adoption of sustainable farming practices: a policy-oriented review. *Eur Rev Agric Econ* **46**, 417-471 (2019).

22. Liu, T., Bruins, R. & Heberling, M. Factors Influencing Farmers's Adoption of Best Management Practices: A Review and Synthesis. *Sustainability-Basel* **10**, 432 (2018).

23. UNECE. Guidance on integrating the environment and climate change in processes for United Nations sustainable development cooperation frameworks. (2021).

24. Snyder, C.S. Enhanced nitrogen fertiliser technologies support the '4R' concept to optimise crop production and minimise environmental losses. *Soil Res* **55**, 463-472 (2017).

25. Gil-Ortiz, R., *et al*. New eco-friendly polymeric-coated urea fertilizers enhanced crop yield in wheat. *Agronomy* **10**, 438 (2020).

26. Chen, D., *et al*. Prospects of improving efficiency of fertiliser nitrogen in Australian agriculture: a review of enhanced efficiency fertilisers. *Soil Res* **46**, 289-301 (2008).

27. Carswell, A., *et al*. Assessing the benefits and wider costs of different N fertilisers for grassland agriculture. *Arch Agron Soil Sci* **65**, 625-639 (2019).

28. Bai, M., *et al*. Lignite effects on NH3, N2O, CO2 and CH4 emissions during composting of manure. *J Environ Manage* **271**, 110960 (2020).

29. Ma, Y., *et al*. Modeling the impact of crop rotation with legume on nitrous oxide emissions from rain-fed agricultural systems in Australia under alternative future climate scenarios. *Sci Total Environ* **630**, 1544-1552 (2018).

30. Rakshit, A., Sarkar, B. & Abhilash, P. *Soil amendments for sustainability: challenges and perspectives* (CRC Press, 2018).

31. Millar, G.D. & Badgery, W.B. Pasture cropping: a new approach to integrate crop and livestock farming systems. *Anim Prod Sci* **49**, 777-787 (2009).

32. Squire, G.R., Quesada, N., Begg, G.S. & Iannetta, P.P. Transitions to greater legume inclusion in cropland: Defining opportunities and estimating benefits for the nitrogen economy. *Food Energy Secur* **8**, e175 (2019).

33. Yigezu, Y.A., *et al*. Legume-based rotations have clear economic advantages over cereal monocropping in dry areas. *Agron Sustain Dev* **39** (2019).

34. Stuart, D., Schewe, R.L. & McDermott, M. Reducing nitrogen fertilizer application as a climate change mitigation strategy: Understanding farmer decision-making and potential barriers to change in the US. *Land Use Policy* **36**, 210-218 (2014).

35. JU, X. & ZHANG, C. Nitrogen cycling and environmental impacts in upland agricultural soils in North China: A review. *J Integr Agr* **16**, 2848-2862 (2017).

36. Pan, B., Lam, S.K., Mosier, A., Luo, Y. & Chen, D. Ammonia volatilization from synthetic fertilizers and its mitigation strategies: A global synthesis. *Agri Eco Environ* **232**, 283-289 (2016).

37. Zhang, X., Mauzerall, D.L., Davidson, E.A., Kanter, D.R. & Cai, R. The Economic and Environmental Consequences of Implementing Nitrogen-Efficient Technologies and

Management Practices in Agriculture. *J Environ Qual* **44**, 312-324 (2015).

38. Johnston, A.M. & Bruulsema, T.W. 4R nutrient stewardship for improved nutrient use efficiency. *Procedia Engineering* **83**, 365-370 (2014).

39. Fixen, P.E. A brief account of the genesis of 4R nutrient stewardship. *Agron J* **112**, 4511-4518 (2020).

40. M U Ller, C., *et al*. Global patterns of crop yield stability under additional nutrient and water inputs. *Plos One* **13**, e198748 (2018).

41. Voss-Fels, K.P., Stahl, A. & Hickey, L.T. Q\&A: Modern crop breeding for future food security. *Bmc Biol* **17**, 1-7 (2019).

42. Ascott, M.J., *et al*. Global patterns of nitrate storage in the vadose zone. *Nat Commun* **8** (2017).

43. Evenson, R.E. & Gollin, D. *Crop variety improvement and its effect on productivity the impact of international agricultural research* (Cabi, 2003).

44. Lenaerts, B., Collard, B.C. & Demont, M. Improving global food security through accelerated plant breeding. *Plant Sci* **287**, 110207 (2019).

45. Vuolo, F., Essl, L. & Atzberger, C. Costs and benefits of satellite-based tools for irrigation management. *Frontiers in Environmental Science* **3** (2015).

46. Dey, N.C., Bala, S.K. & Hayakawa, S. Assessing the economic benefits of improved irrigation management: a case study in Bangladesh. *Water Policy* **8**, 573-584 (2006).

47. Boughlala, M., Gharras, O.E. & Dahan, R. Economic comparison between Conventional and No-Tillage farming systems in Morocco. (2013).

48. Derpsch, R., Friedrich, T., Kassam, A. & Li, H. Current status of adoption of no-till farming in the world and some of its main benefits. *Int J Agr Biol Eng* **3**, 1-25 (2010).

49. Karayel, D. & Sarauskis, E. Environmental impact of no-tillage farming. *Environmental Research, Engineering and Management* **75**, 7-12 (2019).

50. ICID. International Commission on Irrigation & Drainage. (2021).

51. Zhang, X., *et al*. Managing nitrogen for sustainable development. *Nature* **528**, 51-59 (2015).
