# Supplementary material for: Cost-effective mitigation of nitrogen pollution from global croplands
Source: Nature. 2023 Jan 4;613(7942):77–84. doi: 10.1038/s41586-022-05481-8 (PMC9842502; doi:10.1038/s41586-022-05481-8)
Supplement: Supplementary file 2 — Reporting Summary [file 41586_2022_5481_MOESM2_ESM.pdf]

nature portfolio

Corresponding author(s): Baojing Gu

Last updated by author(s): Aug 29, 2022

Reporting Summary

Nature Portfolio wishes to improve the reproducibility of the work that we publish. This form provides structure for consistency and transparency in reporting. For further information on Nature Portfolio policies, see our Editorial Policies and the Editorial Policy Checklist.

Statistics

For all statistical analyses, confirm that the following items are present in the figure legend, table legend, main text, or Methods section.

n/a	Confirmed	
☐	☒	The exact sample size (*n*) for each experimental group/condition, given as a discrete number and unit of measurement
☐	☒	A statement on whether measurements were taken from distinct samples or whether the same sample was measured repeatedly
☒	☐	The statistical test(s) used AND whether they are one- or two-sided<br>*Only common tests should be described solely by name; describe more complex techniques in the Methods section.*
☒	☐	A description of all covariates tested
☐	☒	A description of any assumptions or corrections, such as tests of normality and adjustment for multiple comparisons
☐	☒	A full description of the statistical parameters including central tendency (e.g. means) or other basic estimates (e.g. regression coefficient) AND variation (e.g. standard deviation) or associated estimates of uncertainty (e.g. confidence intervals)
☒	☐	For null hypothesis testing, the test statistic (e.g. *F*, *t*, *r*) with confidence intervals, effect sizes, degrees of freedom and *P* value noted<br>*Give P values as exact values whenever suitable.*
☒	☐	For Bayesian analysis, information on the choice of priors and Markov chain Monte Carlo settings
☒	☐	For hierarchical and complex designs, identification of the appropriate level for tests and full reporting of outcomes
☒	☐	Estimates of effect sizes (e.g. Cohen's *d*, Pearson's *r*), indicating how they were calculated

Our web collection on statistics for biologists contains articles on many of the points above.

Software and code

Policy information about availability of computer code

Data collection	All data were extracted from online database, text or tables directly, or figures using WebPlotDigitizer 4.2.
Data analysis	Data analysis in this study are conducted at Microsoft Excel, MetaWin 2.1, Origin 2022, ArcGIS 10.2, the CHANS, IMAGE, MagPIE, and the GAINS model. Simplified CHANS calculator could be founded at https://person.zju.edu.cn/en/bjgu#930811, GAINS model simulation can be found at https://gains.iiasa.ac.at/models/index.html

For manuscripts utilizing custom algorithms or software that are central to the research but not yet described in published literature, software must be made available to editors and reviewers. We strongly encourage code deposition in a community repository (e.g. GitHub). See the Nature Portfolio guidelines for submitting code & software for further information.

Data

Policy information about availability of data

All manuscripts must include a data availability statement. This statement should provide the following information, where applicable:

- Accession codes, unique identifiers, or web links for publicly available datasets
- A description of any restrictions on data availability
- For clinical datasets or third party data, please ensure that the statement adheres to our policy

The datasets analysed in this study are available from the authors on reasonable request. Correspondence and requests for materials should be addressed to B.G.

and J.X. The literature used in the meta-analysis is listed in Supplementary data S10 in the Excel file. Extended data of the main findings and further discussion can be found in Supplementary Information Section 2.

Human research participants

Policy information about studies involving human research participants and Sex and Gender in Research.

Reporting on sex and gender	not applicable
Population characteristics	not applicable
Recruitment	not applicable
Ethics oversight	not applicable

Note that full information on the approval of the study protocol must also be provided in the manuscript.

Field-specific reporting

Please select the one below that is the best fit for your research. If you are not sure, read the appropriate sections before making your selection.

☐ Life sciences ☐ Behavioural & social sciences ☒ Ecological, evolutionary & environmental sciences

For a reference copy of the document with all sections, see nature.com/documents/nr-reporting-summary-flat.pdf

Ecological, evolutionary & environmental sciences study design

All studies must disclose on these points even when the disclosure is negative.

Study description	This study conducted a global meta-analysis and literature review to select the feasible N mitigation options for croplands with consideration of their impact on NUE and crop yield. A set of tiered scenarios simulation and cost-benefits analysis were then conduced to inform the future mitigation strategy and pathways.
Research sample	The meta-anlysis in this study included 1521 field observations acoss the world during 2000-2020, we identified a group of 11 key measures that can mitigate N losses from croplands for 172 nations/regions.
Sampling strategy	The selection criteria of effective N mitigation options used in this study mianly includes four aspects: high mitigation efficiency; low implementation cost; practical applicability; and synergies or trade-offs. A total of 11 mitigation options for specific cropping systems were included in this study for achieving both agronomic and environmental targets based on the selection criteria.
Data collection	Data used in this study was collected from global and regional statistics, surveys, reports, published papers with field experiment results, and model database (IMAGE, MAgPIE, CHANS model).
Timing and spatial scale	This study did the meta-analysis based on the field experiments conducted in the past two decades (2000-2020) across the world. The global cropland N budgets was compiled on the national scale in 2015. Future tiered scenarios from 2020 to 2050 were simulated to identify each nation's cost-effective mitigation strategy and pathway.
Data exclusions	No data were excluded from the analyses.
Reproducibility	All attempts to repeat the results of this study were successful.
Randomization	This study builds on established models and methods but goes beyond previous research by combining data from FAOSTAT, meta-analysis, and a range of national data sources. This methodology has inevitably simplified the complex N cycling and left out spatial variation. Considering the insufficient understanding and involvement of spatiotemporal heterogeneity in biogeochemical and hydrological processes, using this national-level N budget to explore trajectories of N use and evaluate their environmental impact may lead to biases and uncertainties. However, these uncertainties are systematic rather than random, and therefore do not affect conclusions based on spatial and temporal comparisons.
Blinding	Blinding was not necessary as none of the data used in this study was subjective nor could be influenced by researcher biases

Did the study involve field work? ☐ Yes ☒ No

Reporting for specific materials, systems and methods

We require information from authors about some types of materials, experimental systems and methods used in many studies. Here, indicate whether each material, system or method listed is relevant to your study. If you are not sure if a list item applies to your research, read the appropriate section before selecting a response.

Materials & experimental systems

n/a	Involved in the study
☒	☐ Antibodies
☒	☐ Eukaryotic cell lines
☒	☐ Palaeontology and archaeology
☒	☐ Animals and other organisms
☒	☐ Clinical data
☒	☐ Dual use research of concern

Methods

n/a	Involved in the study
☒	☐ ChIP-seq
☒	☐ Flow cytometry
☒	☐ MRI-based neuroimaging
